# Influence of Exposure and Toxicokinetics on Measures of Aquatic Toxicity for Organic Contaminants: A Case Study Review

**DOI:** 10.1002/ieam.1388

**Published:** 2012-12-10

**Authors:** Peter F Landrum, Peter M Chapman, Jerry Neff, David S Page

**Affiliations:** † 6829 Earhart RoadAnn Arbor, Michigan, USA; ‡ Golder Associates LimitedBurnaby, British Columbia, Canada; § Neff & AssociatesDuxbury, Massachusetts, USA; ‖ Bowdoin College Chemistry Department6600 College Station, Brunswick, Maine 04011-8466, USA

**Keywords:** Dose–response, Petroleum, Mixtures, Absorbed dose, Fish embryos

## Abstract

This theoretical and case study review of dynamic exposures of aquatic organisms to organic contaminants examines variables important for interpreting exposure and therefore toxicity. The timing and magnitude of the absorbed dose change when the dynamics of exposure change. Thus, the dose metric for interpreting toxic responses observed during such exposure conditions is generally limited to the specific experiment and cannot be extrapolated to either other experiments with different exposure dynamics or to field exposures where exposure dynamics usually are different. This is particularly true for mixture exposures, for which the concentration and composition and, therefore, the timing and magnitude of exposure to individual components of different potency and potentially different mechanisms of action can vary. Aquatic toxicology needs studies that develop temporal thresholds for absorbed toxicant doses to allow for better extrapolation between conditions of dynamic exposure. Improved experimental designs are required that include high-quality temporal measures of both the exposure and the absorbed dose to allow better interpretation of data. For the short term, initial water concentration can be considered a conservative measure of exposure, although the extent to which this is true cannot be estimated specifically unless the dynamics of exposure as well as the toxicokinetics of the chemicals in the exposure scenario for the organism of interest are known. A better, but still limited, metric for interpreting the exposure and, therefore, toxicity is the peak absorbed dose, although this neglects toxicodynamics, requires appropriate temporal measures of accumulated dose to determine the peak concentration, and requires temporal thresholds for critical body residue for each component of the mixture. Integr Environ Assess Manag 2013; 9: 196–210. © 2012 SETAC

## INTRODUCTION

Aquatic toxicology has traditionally used the exposure-water concentration of a test substance as an acceptable dose metric, assuming that toxic responses are caused by accumulation of the toxicant at the site of toxic action in the tissues, and that the concentration at the site of toxic action is proportional to the concentration in the exposure water. This assumption is based on a constant exposure concentration and toxicokinetics that do not change over time (McCarty et al. 2011). Laboratory exposures may be acute (usually ≤96 h) or chronic, and vary from static (with or without periodic renewal) to flow-through or to partition-controlled delivery of the test substance (Kiparissis et al. 2003; Rand 2008). However, the assumption that the absorbed dose reflects the water concentration is rarely tested in most experimental designs. The question of representing dose at the site of toxic effect was considered by the 2007 Society of Environmental Toxicology and Chemistry (SETAC) Pellston Workshop, “The Tissue Residue Approach for Toxicity Assessment” (TRA) (Meador et al. 2011). Although there are excellent studies that show that the absorbed organic contaminant concentration is a useful dose metric to describe toxic response, there are complications with most metals, because the total accumulated metal concentration does not always reflect the biologically active metal concentration. The results of this workshop prompted us to review exposure scenarios in toxicity studies where tissue and exposure water concentrations were both measured.

Outside the laboratory, in the aquatic environment, exposures, particularly to low-solubility organic chemicals, are rarely continuous and often are pulses with varying rates of decay (Reinert et al. 2002). Assessment of aquatic toxicity from discontinuous or fluctuating exposures requires, at a minimum, knowledge of the toxicokinetics and toxicodynamics of the substance in the test organism (Hickie et al. 1995; Jager et al. 2011). Toxicokinetics, in this context, describes the rates of inward and outward flux of the substance between the exposure water and the tissues of the test organism and the physical, chemical, and biological factors, including biotransformation, that control the uptake and release rates. Toxicodynamics describes the interactions of the absorbed substance and the sites of toxic action that result in biochemical and physiological effects in the organism (Jager et al. 2010). Models of such exposures, developed for both acute (Jager et al. 2011) and chronic (Ashauer et al. 2011) effects on aquatic organisms, also require an understanding of carry-over toxicity that results from incomplete recovery in pulsed exposures (Ashauer et al. 2010) and the effects of mixtures of chemicals with different toxicokinetics on the expression of different biochemical responses (Jager et al. 2010). The data required for such models are typically not collected during most aquatic toxicity testing. In fact, most such testing assumes, but does not demonstrate, constant water concentrations, and body residues are not typically measured.

Laboratory toxicity testing can be conducted either with or without consideration of environmental conditions, depending on the objectives of the testing. Tests designed to elucidate mechanisms of toxicity of a chemical often use unrealistic exposure methods and concentrations, such that the results are difficult to extrapolate to the field (Hinton et al. 2005). Laboratory toxicity tests intended to assess potential health risks to aquatic organisms and ecosystems from release of a chemical or mixture of chemicals usually seek to mimic the conditions of exposure occurring in the environment. Following an aquatic oil spill, for example, local aquatic organisms and biological communities are at risk of exposure to an extremely complex mixture of petroleum hydrocarbons and related organic compounds that varies in time and space in both composition and concentration. Exposure of aquatic organisms to toxic fractions of the spilled oil may be via water, food, and sediments and for semiaquatic species, such as birds and mammals, from air (Neff et al. 2011). For example, laboratory toxicity testing of oil can attempt to simulate exposure to petroleum hydrocarbons in water leaching from oiled shoreline sediments by using effluents from oiled-gravel columns (Carls et al. 1999, 2005; Heintz et al. 1999; Carls and Thedinga 2010; Frantzen et al. 2012) or to oil in the water column using laboratory-prepared water accommodated fractions (WAF) of the spilled oil (Anderson et al. 1974; Girling et al. 1994; Clark et al. 2001; Wu et al. 2012). This article evaluates these 2 environmental exposure conditions, specifically an open system (flow through) and a closed or static system where exposure concentrations are changing (Case Study 1 and Case Study 2, respectively), relative to the appropriate measurements required to attribute observed toxicity to specific contaminant exposures. Although we had an appropriate example for the open-exposure system in Case Study 1 that allowed examination of a complex organic mixture from petroleum exposures, we could not find a WAF study that measured both the exposure concentration and tissue residue for the static or closed system. However, we were able to find an excellent study using another nonpolar contaminant, dieldrin. Although not a complex mixture and not a petroleum hydrocarbon, the selected study does demonstrate the exposure principles for aquatic organisms exposed to organic contaminants under static conditions, which would be applicable to the individual components in WAF studies.

We examine the consequences of changing concentrations and compositions of complex mixtures in water on the exposure and dose metric for toxicological studies. We begin with descriptions of the toxicokinetics for different theoretical scenarios to illustrate problems, and solutions, for determining the toxicological dose metric, comparing exposures (concentrations in water) and doses (concentrations in tissue). We then build on these theoretical scenarios with the above 2 case studies. Specific recommendations and critical further research needs are provided based on both the theoretical scenarios and the case studies.

Surprisingly, we had great difficulty in finding studies with appropriate data sets for both water exposures and tissue doses for the purposes of this article. Many articles on the aquatic toxicology of petroleum, for example, provide data on the initial concentration of individual and total hydrocarbons in exposure water but do not provide data on the tissue concentrations, or do not provide sufficient data on temporal changes in hydrocarbon concentrations in exposure water and tissues that conclusions about toxicokinetics and toxicodynamics can be ascertained. Future studies need to provide, in addition to temporal data on water quality parameters (e.g., temperature, pH, conductivity, salinity, dissolved O_2_), comparable data on temporal patterns of exposure concentrations and temporal patterns of accumulation of the target chemicals in tissues of the test organisms. Although we provide a critical evaluation of the 2 case studies, we also congratulate the authors of those studies for providing more data and information for analysis than other articles that evaluated toxicological responses to time-varying concentrations of individual compounds or complex mixtures.

## THEORETICAL SCENARIOS

The influence of changing exposure concentration on the absorbed dose that an organism experiences is influenced by 2 main sets of rate processes: 1) those that govern the pattern and rate of change of the exposure concentration, and 2) those that govern the dose, measured as the rate of accumulation and loss of the contaminant and/or contaminants by the organism. Each of these 2 rate processes is controlled by different parameters. The rate of change in exposure concentration depends on source fidelity, hydrologic conditions in exposure chambers (or field site), and physical–chemical characteristics of the compounds in the mixture (particularly aqueous solubility and volatility). The uptake rate of each chemical in the mixture from exposure water by the organism depends on the physical–chemical characteristics of the compound that define its forms in the exposure medium and relative affinity of the compound for water and tissues, the relative absorptive surface-to-volume ratio of the organism, and the capability of the organism to eliminate the compound from its tissues by passive or active means (diffusion and biotransformation/excretion). The balance of these 2 sets of rate processes will determine how rapidly a substance is accumulated, the peak tissue concentration and timing of that peak tissue concentration, and the rate at which the substance is eliminated. To demonstrate some of these characteristics, simple scenarios were created that mimic the early declining exposure conditions for a test substance in aquatic toxicity tests. Specifically, the concentration of the substance in exposure water was assigned a simple first order decay rate, uptake and elimination rate constants for the organism were fixed, and biotransformation was implicitly included in the elimination rate constant. The toxicokinetics for the organism are first order and fit Equation 1


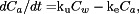
(1)

where *C*_*a*_ is the concentration in the organism, *C*_*w*_ is the concentration in the water, *t* is time (d, days) k_u_ is the uptake clearance (uptake rate constant) by the organism from water (mL g^−1^ d^−1^), and k_e_ is the total elimination-rate constant (d^−1^). The actual units employed depend on the choice of the modeler but are kept compatible for volume, mass of compound, organism mass, and time (Landrum et al. 1992).

### Influence of the rate of decline of water concentration on observed accumulation using fixed toxicokinetics

**Figure 1:**
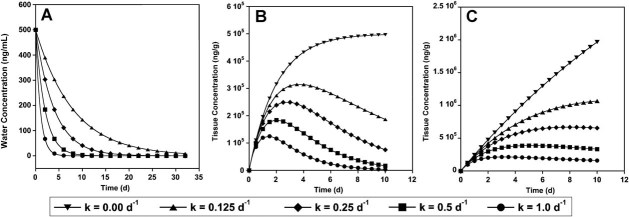
First order decay of an initial water concentration of 500 ng mL^−1^ for different loss-rate constants given in the figure (**A**). Uptake curves calculated using Equation 1 for an organism exposed to the same initial water concentration of 500 ng mL^−1^ and varying rates of first order decay (see figure) with constant toxicokinetics (uptake-rate constant (k_u_) = 500 mL g^−1^ d^−1^; elimination-rate constant (k_e_) = 0.5 d^−1^) (**B**). Bioaccumulation of a substance during exposure to the same initial water concentration as in (A) with different rates of decay for the water concentration (**C**). The organism uptake-rate constant (k_u_) is 500 mL g^−1^d^−1^ as in (B), but the elimination-rate constant (k_e_) is set at 0.05 d^−1^, slower than the rate of decay for the water.

A scenario was created in which an initial water concentration of a test substance is fixed at 500 ng mL^−1^ and the rate of loss of the substance from water varies from 0 to 1.0 d^−1^. Figure 1A shows the effect of the varying loss rate of the substance from the water on the overall decline in water concentrations (*C*_*w*_ in Eqn. 1) with time. For the parameters in Equation 1, the uptake and elimination rate constants are set at 500 mL g^−1^ d^−1^ and 0.5 d^−1^, respectively. The absorbed dose shows 2 important characteristics (Figure 1B). First, the magnitude of the peak tissue concentration declines as the rate of loss from the water increases. Second, the time to peak tissue concentration from the initiation of exposure declines with increasing rate of loss of the substance from water. What this means is that organisms exposed to the same initial water concentration will experience different absorbed doses as the rate of decline of substance concentration in exposure water changes, without invoking any changes in the organism toxicokinetics. Thus, any attempt to compare data among exposures with declining water concentrations needs to ensure that the declines in water concentration are the same or the comparison cannot be readily made. The initial water concentration is a conservative estimate of exposure, but the extent to which this is the case will vary depending on the rate of decline of the water concentration. The initial water concentration will not likely be a useful measure of exposure for dose–response across a series of treatments unless it can be demonstrated that the loss rate from the water is the same, or nearly so, for each exposure concentration.

This simple scenario demonstrates that the peak tissue concentration varies in magnitude and time to attainment with changing substance loss rates in exposure water. Thus, interpreting the toxicity of a substance based on the peak tissue concentration can be misleading and may be problematic, depending on exactly what endpoint is investigated. For instance, the uptake rate and time to reach critical toxicant concentrations in fish embryo tissues relative to the timing of embryonic-development events determines the types of sublethal effects observed and their severity; a simple dose–response may not occur (Rosenthal and Alderdice 1976; McIntosh et al. 2010). Even if the endpoint was simply mortality of adults, the peak concentration might not be an appropriate exposure metric as it would not account for toxicodynamics and organism-repair processes. Such issues become more problematic when considering not just a single substance, but complex mixtures of chemicals with different concentrations and associated dynamics of exposure, toxicokinetics, toxicodynamics, potencies, and potentially different mechanisms of action.

### Influence of toxicokinetics including uptake, elimination, and biotransformation on tissue accumulation

The original scenario shown in Figure 1B was adjusted by reducing the elimination rate constant in Equation 1 10-fold to 0.05 d^−1^ (Figure 1C). The time to peak tissue concentration is now lengthened relative to the more rapid elimination in the original scenario (Figure 1B) such that, at the lowest rate of loss of the substance from exposure water, the peak tissue concentration does not occur during the 10-d exposure period (Figure 1C). In this amended scenario, toxicokinetics appear sufficiently similar to constant concentration water exposures, except in magnitude, that tissue data could be interpreted with more reliability, and timing for biological changes would likely not be as critical. However, as with the original scenario, the initial water concentration, whereas a conservative representation of the organism exposure, would still not address the impact of different dynamic conditions on the magnitude of accumulated dose. The initial water concentration is still not an acceptable measure of exposure unless the exposure dynamics are essentially the same among treatments.

### Does the use of time-weighted average or geometric mean concentration appropriately represent exposure?

**Figure 2:**
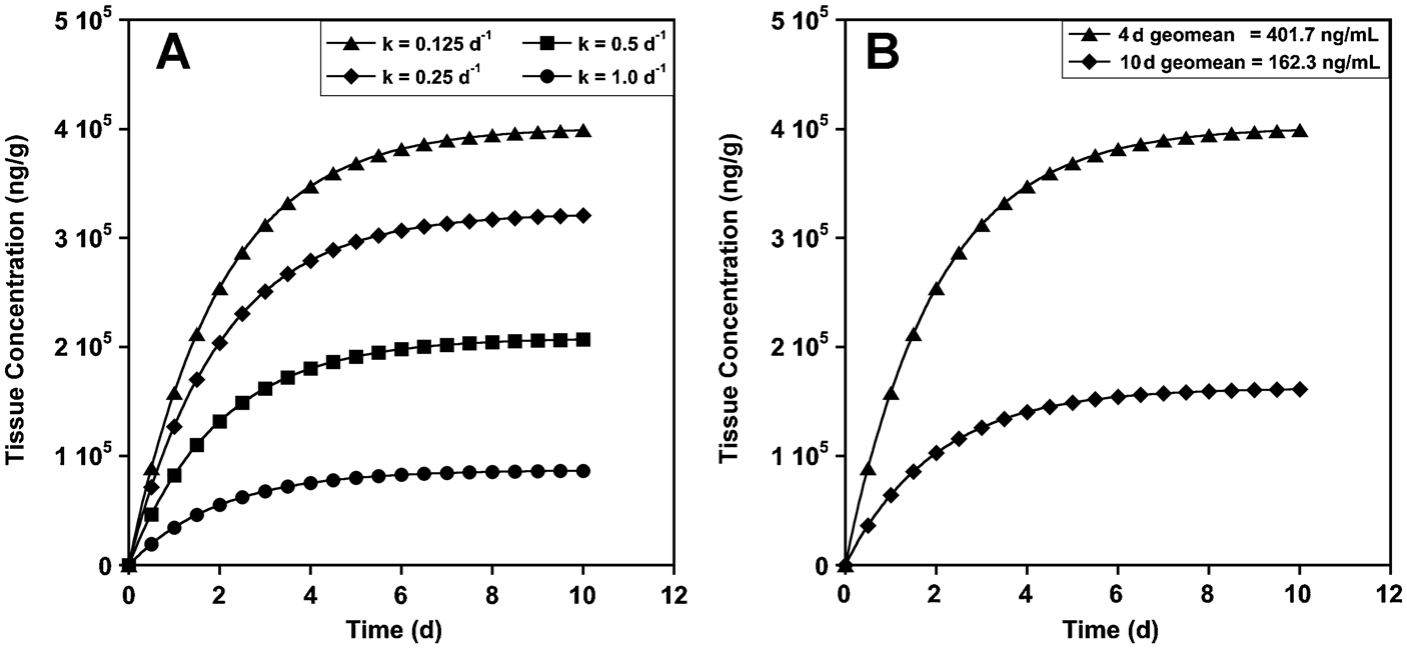
Bioaccumulation kinetics using fixed toxicokinetic parameters equivalent to Figure 1B but using the geometric mean of the day 0–day 4 water concentrations as the assumed water concentration at all time points (**A**). Bioaccumulation based on a constant exposure concentration represented by the geometric mean concentration over 4 and 10 d using the toxicokinetics of Figure 1B for the slowest decline in water concentration (0.125 d^−1^) and thus the one with the largest geometric mean (**B**).

The above 2 scenarios lead to the question of whether some integration of water concentrations can represent the organism exposure. The geometric mean has been suggested as a suitable representation for the exposure of organisms to temporal changes in concentration of the substance in exposure water (Carls and Thedinga 2010). To investigate this question, a third scenario (Figure 2) was created that has the same toxicokinetics as the original Figure 1 scenario (k_u_ = 500 mL g^−1^ d^−1^, k_e_ = 0.5 d^−1^). However, exposure is represented as a constant concentration, estimated as the geometric mean of the first 4 days of exposure water concentrations (Figure 2A). When comparing Figure 1B and Figure 2A, it is clear that the organism is experiencing a much different exposure when the constant geometric mean water concentration is used, rather than when the actual time-variable water concentration is used to define exposure. Estimated tissue concentrations after a 10-d exposure, based on a 4-d geometric mean water concentration, are approximately 2 times greater than 10-d tissue concentrations in the original declining exposure concentration scenario (Figure 1B). However, after a 4-d exposure, estimated tissue concentrations are only slightly higher for the 4-d geometric mean exposure concentrations than for the declining concentration exposures at 4 d. For example, the predicted concentrations in the organism following exposure for 4 or 10 d to an initial concentration of 500 ng/mL, which underwent a first order decay at a rate of 0.125/d, are approximately 320 000 and 190 000 ng/g, respectively (Figure 1B). The 4-d geometric mean concentration in this exposure scenario is 401.7 ng/mL and gives an estimated tissue concentration following 4 and 10 d of exposure of 350,000 and 400,000 ng/g, respectively (Figure 2A). The time required to reach maximum tissue concentration is greater when geometric mean exposure concentration rather than the actual declining water concentration is used. This means that the geometric mean exposure concentration was a reasonable metric for exposure at the end of the 4-d period included in the geometric mean, where the overall concentration change was less than a factor of 2 (1 half-life), but it was not suitable for estimating exposure beyond the averaging period.

The time range of concentrations used to determine the geometric mean can influence apparent exposure represented by the geometric mean. Extending the use of measured concentrations out to 10 d in calculating a 10-d geometric mean (Figure 2B) creates an exposure in which the steady-state tissue concentration is substantially lower than the peak concentration estimated from declining exposure concentrations. Thus, using a geometric mean to represent exposure water concentration does not take into consideration the dynamics of the water or toxicokinetics and can provide misleading information on organism exposure to a substance. An even larger discrepancy would result if the geometric mean were based on data taken beyond the time period in which a toxicity response was measured. Geometric means also do not represent the dynamics of variable exposures. Thus, the use of geometric means to represent aqueous exposure conditions should be used with extreme caution except possibly if the data points comprising such means have similar values. The acceptable range of concentrations for application of a geometric mean requires comparisons between the rate of change of the water concentrations and toxicokinetics to determine the conditions under which such averaging is appropriate.

### Summary

Although the above scenarios represent the likely combination of conditions that might be expected and would need to be considered for variable water concentrations, the full scope of such comparisons has not been explored. Different exposures with different water and toxicokinetics remain to be considered as do complex mixtures containing components with different toxicokinetics, toxicodynamics, and potencies. The complexity of such exposures suggests that some simplifications will be needed in the short term that can address environmental exposures conservatively while still allowing extrapolation beyond specific experiments.

## CASE STUDY 1: DECLINING DOSE EXPOSURE—HERRING EGGS/LARVAE AND OILED-GRAVEL COLUMNS

In the experiments described by Carls et al. (1997, 1999), Pacific herring (*Clupea pallasi*) eggs were exposed to seawater effluents from artificially weathered crude oil coated onto gravel and placed in columns through which seawater was pumped. The exposures comprised 2 experiments: a 16-d exposure of herring embryos to effluents from the freshly prepared oiled-gravel columns, called “less weathered oil” (LWO), followed by a 13-d period when water flow through the columns was turned off, and then a 16-d exposure of a different set of herring embryos to effluents from the same oiled-gravel columns, called “more weathered oil” (MWO), was initiated. There were 4 treatments in each experiment: High, Mid (middle), Low, and Trace oil loading to the gravel along with a control.

The toxicity of the effluents was evaluated based on embryo and larval mortality and 10 sublethal toxicity parameters (Carls et al. 1999). Total polycyclic aromatic hydrocarbons (PAH) concentrations in water, measured at the beginning of each experiment, served as the selected dose metric. Tissue PAH residues were measured in embryos at different times during exposure, but not for all treatments or at the same frequency in all exposures. Water PAH concentrations were measured at 6 to 8 time intervals for different treatments of the LWO experiment and at 5 time intervals for the MWO experiment treatments, except controls. Tissue PAH concentrations also were measured at 3 time intervals during the exposure period for only the LWO-High and LWO-Control treatments and at 3 to 5 time intervals during the exposure period for all MWO treatments. Tissue PAH concentrations were also measured at 4 time intervals postexposure for the LWO-High and 3 time intervals postexposure for the LWO-control. No postexposure data were collected for the MWO experiment. PAH concentrations were not measured in herring embryos from the LWO-Mid, Low, and Trace treatments (EVOSTC 2009).

As demonstrated in the theoretical discussion, the initial total PAH (TPAH) concentration in water provides a conservative description of exposure; however, peak tissue dose will vary in time and magnitude depending on the initial water concentration, rate of loss of different target PAH from the water, and the toxicokinetics of each PAH in the organism. In the case of TPAH, as shown below, exposure dynamics also depend on the composition of the mixture. Thus, selecting initial water concentrations of TPAH as a dose metric may not be an accurate or appropriate approach for assessing dose (Landrum et al. 2012) and would not be appropriate for comparisons among experiments or spill sites unless the loss rate and mixture compositions in exposure water were identical or at least very similar. The extent of conservatism will depend on the actual kinetics of the decline in water concentrations, the composition of the mixture, and the toxicokinetics of the different mixture components in the organism.

We investigated the Carls et al. (1997, 1999) experiments based on toxicokinetics and simulations that included treatments where no tissue concentration measurements were taken to better interpret the exposure conditions represented by the treatments. Three analyses were performed for unsubstituted PAH, alkylated PAH, and TPAH. In the first analysis, because of variability in the wet to dry weight data (EVOSTC 2009), toxicokinetics were explored to assess the significance of this variability to further analyses. The data were fit to a first order toxicokinetic model (Eqn. 1) by numerical integration using Scientist (Micromath, St Louis, MO). The model included changes in water concentration with cessation of exposure after 16 d. This first order equation assumes that the elimination rate constant includes both passive loss from the organism and loss due to biotransformation.

In the second analysis, simulations of bioaccumulation were carried out based on toxicokinetics determined by Mathew et al. (2008), using data from Carls et al. (1999) and using the best representation of the change in water concentration from the data (EVOSTC 2009). The toxicokinetic parameters determined by Mathew et al. (2008), where the loss rate constant due to passive elimination based on partitioning and the biotransformation rate constant, were combined to provide the total loss rate constant. Our simulation results were compared to measured tissue concentrations from the same data source.

In the third analysis, because the work of Mathew et al. (2008) did not include TPAH as part of the analysis, the LWO-High treatment data (*n* = 10), for which both water and tissue concentrations were measured, were fit to a first order toxicokinetic model (Eqn. 1). Kinetic constants were used to simulate the other treatment concentrations for TPAH bioaccumulation.

The modeling analyses involved TPAH, naphthalene, C1(mono-alkyl)-naphthalenes, phenanthrene, C1-phenanthrenes, dibenzothiophene, C1-dibenzothiophenes, chrysene, and C1-chrysenes. These PAH covered the full range hydrophobicity modeled by Mathew et al. (2008).

### PAH concentrations in exposure water

Concentrations of individual and TPAH in exposure water showed different trends over time, ranging from little or no change during the 16-d exposure period to double exponential declines. Most of the data sets followed a single first order decay model (Table 1). However, for naphthalene and C1-naphthalenes, none of the models was adequate for describing trends in water concentration over time. For these 2 compounds, water concentration trends typically showed declines that were best described by a first order decay model for 2 individual time periods, each of which was used to model contaminant uptake by the herring eggs.

**Table 1:** Models for the change in water concentration (ng/mL) over the 16-d exposure duration for the 6 treatments examined for toxicokinetics from the data of Carls et al. (1999) as found in EVOSTC (2009) a

Compound	LWO-High^b^	LWO-Mid^b^	LWO-Low^b^	MWO-High^b^	MWO-Mid^b^	MWO-Low^b^	MWO-Trace^b^
Naphthalene	0–4 d; 10.7e^−0.67*t*^	1.37e^−0.641*t*^	0.211e^−0.32*t*^	0.028e^−0.88*t*^ + 0.015e^−0.11*t*^	0.0051 ± 0.0008	0.0098e^−0.045*t*^	0.00968e^−0.046*t*^

	4–16; d 0.47e^−0.15*t*^						

C1-naphthalenes	0–2 d; 40.2e *−* *0.76*t	10.0e^−0.29*t*^	0.50e^−0.20*t*^	0.56e^−0.43*t*^	0.0067e^−0.031*t*^	0.012e^−0.043*t*^	0.014e^−10.06*t*^

	2–16 d; 8.2 ± 2.0						

Phenanthrene	1.38e^−0.033*t*^	1.14–0.72*t*	0.17e^−1.57*t*^ + 0.14e^−0.21*t*^	0.18e^−0.30*t*^	0.0035 ± 0.00023	0.0086e^−0.16*t*^	0.0036e^−0.055*t*^

C1-phenanthrenes	1.04 ± 0.14	1.08–0.055*t*	0.74e^−0.22*t*^	0.40e^−0.20*t*^	0.016e^−0.029*t*^	0.0084e^−0.25*t*^	0.0025–0.00019*t*

Dibenzothiophene	1.09e^−0.03*t*^	0.86–0.054*t*	0.11e^−1.82*t*^ + 0.089e^−0.23*t*^	0.22e^−0.20*t*^	0.0011 ± 0.00042	0.00097e^−0.047*t*^	0.0011e^−0.059*t*^

C1-dibenzothiophenes	5.02 ± 0.074	0.536–0.0254*t*	0.33e^−0.22*t*^	0.12e^−0.14*t*^	0.011e^−0.03*t*^	0.0054e^−0.15*t*^	0.0024e^−0.11*t*^

Chrysene	0.010 ± 0.003	0.0.011 ± 0.0041	0.0096e^−0.036*t*^	0.015 ± 0.0026	0.0093 ± 0.0011	0.0064e^−0.017*t*^	0.0031e^−0.065*t*^

C1-chrysenes	0.010 ± 0.006	0.0073 ± 0.0020	0.0069e^−0.031*t*^	0.013 ± 0.0040	0.0058 ± 0.0010	0.0073e^−0.028*t*^	0.0040e^−0.023*t*^

TPAH	51.3e^−1.14*t*^ + 34.7e^−0.02*t*^	33.7e^−0.15*t*^	8.20e^−0.027*t*^	7.53e^−0.17*t*^	0.74e^−0.021*t*^	0.40e^−0.12*t*^	0.11e^−0.057*t*^

PAH = polycyclic aromatic hydrocarbons.

a Data can be represented as average ± SD, as a single exponential in the form *C*_*w*_ = *C*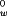 e^−*kt*^, or as a double exponential *C*_*w*_ = *C*
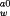
e^−*k1t*^ + *C*
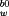
e^−*k2t*^. *r*^2^ values for the fits ranged from 0.999 to 0.939 except for C1-phenanthrene (*r*^2^ = 0.699)

b Less weathered oil (LWO) and more weathered oil (MWO) as defined by Carls et al. (1999). Mid represents the middle gravel-load concentration among the treatments.

Trends in declining water TPAH concentrations, used by Carls et al. (1999) as the representative dose metric, were dominated by the rates of decline for the lower molecular weight compounds, particularly for the LWO-High treatment. Concentrations of higher molecular weight compounds, such as chrysene, remained nearly constant during the tests. The traces of 4- through 6-ring PAH in the exposure water are likely associated almost exclusively with micro-oil droplets (Faksness et al. 2004; Redman et al. 2012) that have a PAH composition similar to that in the oiled-gravel columns. These PAH are depleted from the oiled gravel columns slowly, because of their very low aqueous solubilities and high log *K*_*ow*_values (Schleup et al. 2002), explaining their low, but relatively stable concentrations in the exposure water.

### Wet versus dry weight for modeling

Because there was substantial variability in the embryo tissue dry/wet weight ratio in the Carls et al. (1999) herring embryo study (EVOSTC 2009), the appropriate condition for modeling the toxicokinetics was in question. The LWO-High 4-d tissue sample had the lowest dry/wet weight ratio (0.06 = 94% water); the ratio for embryos from all other exposure concentrations ranged from 0.12 to 0.19, suggesting that the relationship was otherwise not extremely variable. The average dry/wet weight ratio for embryos from all exposure concentrations and sampling times, except control, was 0.146 ± 0.027 (mean ± SD, *n* = 25).

The comparison between modeling the kinetics using wet or dry weight was based on the LWO-High data set, which had the most data as well as the highest variation in dry/wet weight ratio. TPAH was a useful data set to model because it contained the most detailed data for the series of treatments and provided the kinetics for later use for simulations.

**Figure 3:**
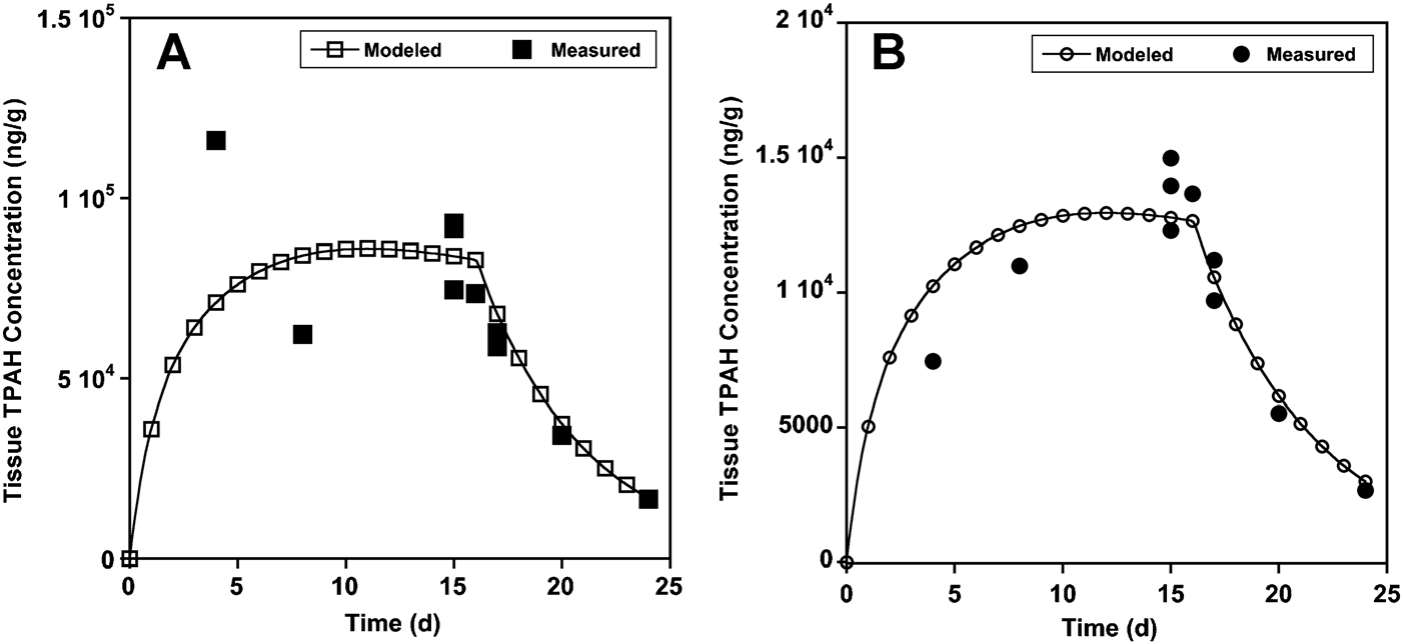
Model of the toxicokinetics of accumulation of TPAH for the LWO-High treatment on a dry weight (**A**) and wet weight basis (**B**) using data taken from EVOSTC (2009).

The model (Eqn. 1) for the toxicokinetics of TPAH on a dry weight basis yielded a coefficient of determination (*r*^2^) of 0.95 with a k_u_ of 616.5 ± 49.4 mL g^−1^_dry weight_ d^−1^ and a k_e_ of 0.20 ± 0.01 d^−1^ (Figure 3A). On a wet weight basis, the *r*^2^ was 0.98 with a k_u_ of 85.4 ± 13.1 mg g^−1^_wet weight_ d^−1^ and a k_e_ of 0.18 ± 0.03 d^−1^ (Figure 3B). The k_e_ values yielded similar elimination half-lives of 3.4 to 3.8 d for the 2 determinations. The 2 uptake constants only differed by a dry/wet weight ratio of 0.139, which fit within the range of most of the data, and which was not statistically different from the average across all treatments. Based on the similarity of the data fits, toxicokinetics were evaluated on a wet weight basis as per Mathew et al. (2008).

### Evaluating toxicokinetics for consistency across treatments

If all treatments had adequate tissue data available for comparison, the toxicokinetics for each treatment could have been determined and a comparison of the exposure conditions across treatments could have been easily assessed. Unfortunately, the treatments LWO-Mid, -Low, and -Trace did not have any measured tissue data, and there were few and inconsistent data points for the MWO treatments (EVOSTC 2009).

However, assuming that the toxicokinetics did not vary across treatments, it was reasonable to apply the toxicokinetic parameters from Mathew et al. (2008) to each treatment to represent the toxicokinetics across all treatments (J McGrath, HydroQual, Mahwah, NJ, USA, personal communication). The kinetic constants from Mathew et al. (2008) for specific compounds were fit using available water data (Table 1). Naphthalene, C1-naphthalenes, phenanthrene, C1-phenanthrenes, dibenzothiophene, C1-dibenzothiophenes, chrysene, and C1-chrysenes were examined. Of these, naphthalene, C1-naphthalenes, and C1-chrysenes could not be fully examined because problems were identified with the toxicokinetics constants provided by Mathew et al. (2008). Specifically, problems were found with the elimination rate constant for naphthalene and C1-naphthalenes and both the uptake and elimination constants for C1-chrysenes. In the case of the naphthalenes, the reported elimination constants were substantially larger than those found by fitting the LWO-High naphthalene and C1-naphthalene data independently; this likely is the result of the finding in Mathew et al. (2008) that the rate constant determined for metabolism was negative, indicating that their model was incorrect for these 2 compounds.

To correct for this issue, the elimination constants for naphthalene and C1-naphthalene were determined by fitting the LWO-High data independently to provide the best estimate for their elimination rate constants. Fitting these data independently led to estimates for the uptake constants that were essentially the same as the Mathew et al. (2008) published values (1.03 ± 0.05 L g^−1^ lipid d^−1^ for naphthalene and 10.0 ± 1.8 L g^−1^ lipid d^−1^ for C1-naphthalenes). Statistical significance could not be determined because Mathew et al. (2008) did not publish the estimates for the error terms for the rate constants. However, because the values were similar, the uptake constant published by Mathew et al. (2008) was retained for modeling and the estimate of the elimination from the independent model was substituted (Table 2).

**Table 2:** Toxicokinetic parameters used for modeling tissue uptake^a^

Compound	k_u_ (L g^−1^ lipid d^−1^)	k_e_ (d^−1^)^b^
Naphthalene	0.99	0.158 ± 0.008^c^

C1-naphthalenes	7.39	0.252 ± 0.046^c^

Dibenzothiophene	5.21	0.22

C1-dibenzothiophenes	4.34	0.27

Phenanthrene	9.69	0.36

C1-phenanthrenes	5.48	0.28

Chrysene	6.73	0.26

C1-chrysenes	0.518 ± 0.074^c^	0.133 ± 0.046^c^

TPAH	1.15 ± 0.178^c^	0.18 ± 0.029^c^

a From Mathew et al. (2008) except where noted. k_u_ is the uptake rate constant and k_e_ is the elimination rate constant.

b The elimination-rate constant is the sum of the passive elimination rate constant k_r_ and the metabolism rate constant k_m_ found in Mathew et al. (2008).

c Values taken from fits to the data using Equation 1 for the LWO-High treatment. See text for more details.

For the C1-chrysenes, both the uptake and elimination rates of Mathew et al. (2008) were too large compared to an independent fit of the C1-chrysene data for LWO-High. Their published values resulted in model simulations of the kinetics that grossly overestimated the measured values. This discrepancy may have resulted from the approach used to model the water concentration, which varied from an initial estimated value of 3.3 ng L^−1^ to values as high as 20.76 ng L^−1^. As discussed above, most of the C1-chrysenes (and other high log *K*_*ow*_ PAH) were likely associated with microdroplets in the exposure water, with very little in the more bioavailable dissolved form, perhaps explaining the unrealistic uptake rates. We elected to estimate the water concentration as a constant using the average concentration, which may have been different from the approach that Mathew et al. (2008) selected to model their water concentrations. To allow for model comparison to measured data, we employed both the uptake and elimination rate constants (Table 2) determined by fitting the uptake to the first order model from the LWO-High C1-chrysenes data (EVOSTC 2009).

**Figure 4:**
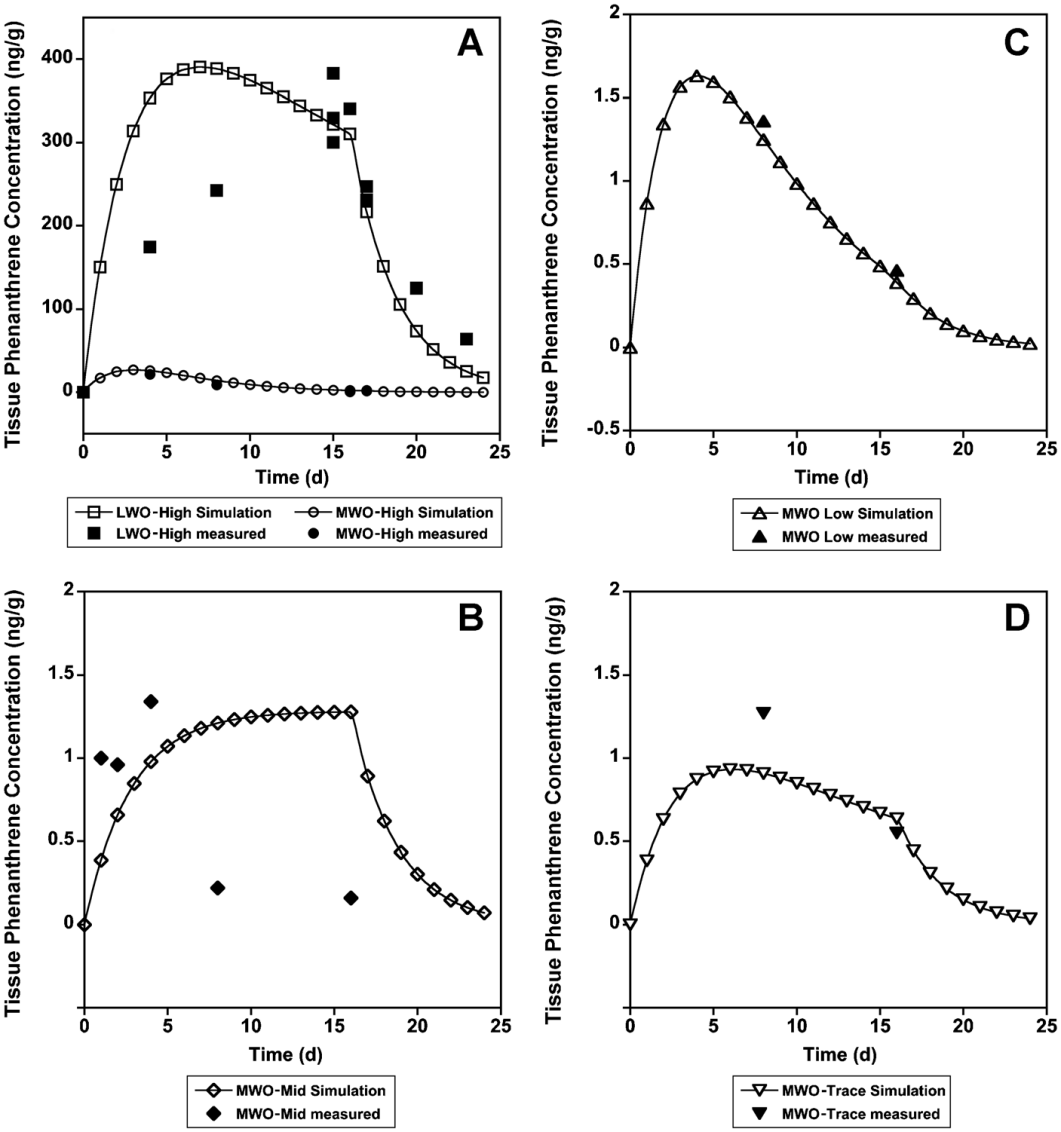
Comparison of the toxicokinetics model with measured tissue data (EVOSTC 2009) for phenanthrene across treatments (**A–D**).

**Figure 5:**
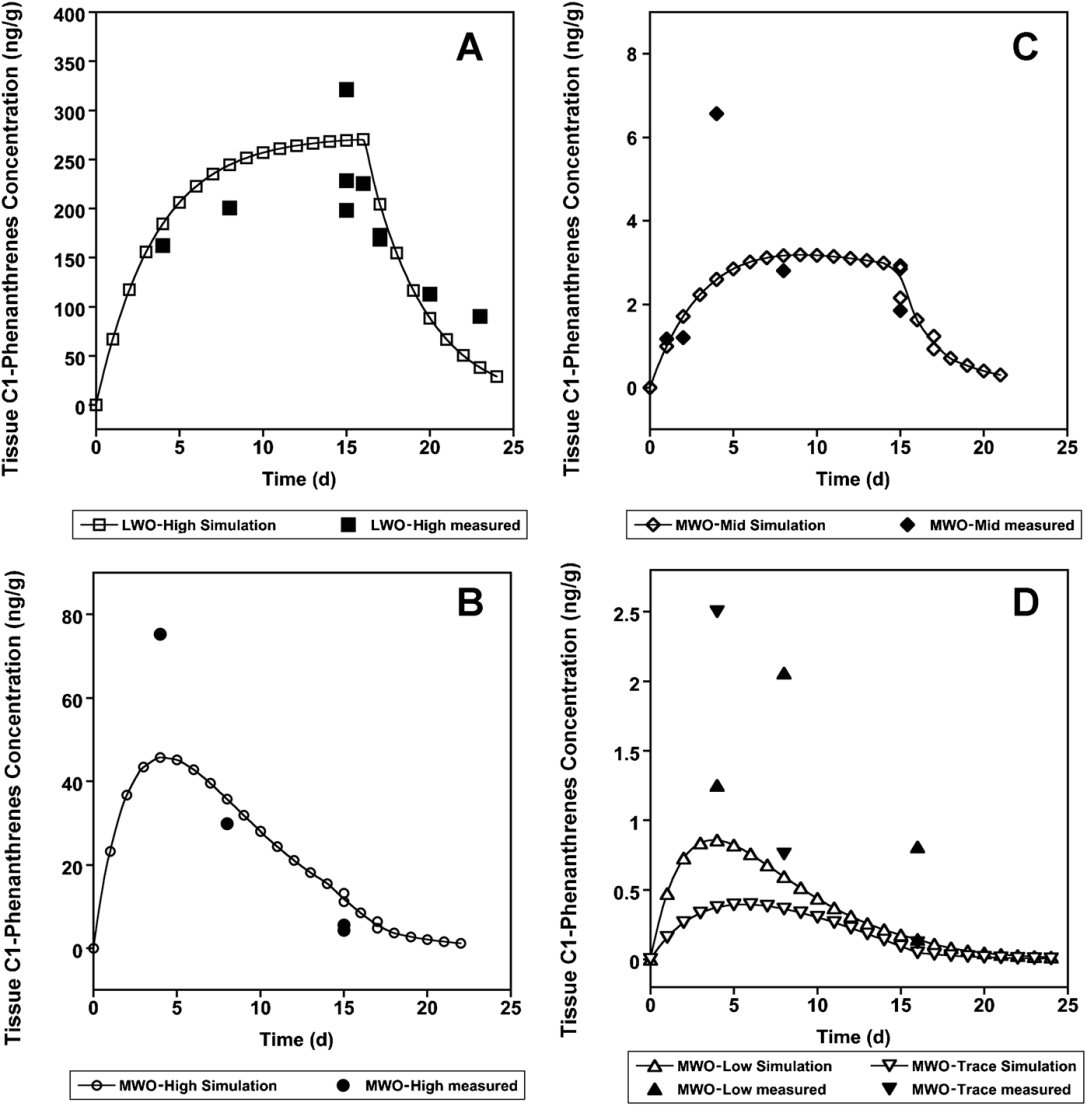
Comparison of the toxicokinetics model with measured tissue (EVOSTC 2009) data for C1-phenanthrenes across treatments (**A–D**).

Total elimination rate used for the modeling was the combination of the loss from passive elimination rate (k_r_) and biotransformation rate (k_m_) (Mathew et al. 2008). Consistency across treatments was evaluated using the model data for phenanthrene (Figure 4), C1-phenanthrenes (Figure 5), dibenzothiophene (Supplemental Figure 1), and C1-dibenzothiophenes (Supplemental Figure 2). These data represent both a parent and an alkylated PAH. It was important to examine the alkylated PAH as well as the unsubstituted PAH because the alkylated PAH may be more potent for some of the endpoints (Rhodes et al. 2005; Turcotte et al. 2011).

In general, modeled data agree with the empirical data. The relative percentage differences (RPDs) between the modeled and measured data were 46.9 ± 43.7% for phenanthrene and 50.4 ± 40.6% for C1-phenanthrenes. The RPDs for dibenzothiophene and C1-dibenzothiophenes were 38.6 ± 24.4% and 33.9 ± 30.8%, respectively. These comparisons indicate that the modeled data and empirical data were comparable within about a factor of 2, indicating that the model was robust, and that estimates of the toxicokinetic parameters did not vary across the treatments for individual compounds. This finding supports the hypothesis that a simulation of the toxicokinetics, particularly where there were no measured tissue values, could estimate absorbed doses for similar exposure conditions, allowing tissue residue evaluation across all treatments. The toxicokinetics for naphthalene, C1-naphthalenes, chrysene, and C1-chrysenes (Supplemental Figures 3–6) did not fit the model as well, because of the complications with the toxicokinetics parameters discussed above.

The toxicokinetics determined from the LWO-High data set for TPAH were used for modeling across all treatments. The comparison of the TPAH across treatments was generally not as good as for the individual PAH discussed above and tended to show greater deviation at the lower concentrations for the MWO treatments (Supplemental Figure 7). The fit of the TPAH to obtain toxicokinetic parameters for modeling was dominated by naphthalene and C1-naphthalenes for LWO-High, possibly explaining a general overprediction of accumulation for the MWO treatments where these compounds represented a lower proportion of the water concentration.

Analytical data variability was assessed relative to the modeling results. There were few replicate measurements and only 1 triplicate tissue PAH concentration measurement (EVOSTC 2009) in the Carls et al. (1999) study. The triplicate tissue residue measurement on day 15 of the LWO-High treatment allowed for calculation of percentage standard deviation (PSD), which ranged from 5.8% for naphthalene to 30.9% for chrysene with an average of 16.9 ± 7.8% for the PAH selected for evaluation, including TPAH. The RPD determined for duplicate tissue measurements ranged from 0.6% for chrysene to 39.1% for naphthalene with an average of 10.5 ± 11.8%. Variability increased as the TPAH concentration declined. In the MWO-High treatment the RPD ranged from 5.6% for chrysene to 113.1% for naphthalene with an average across the substances examined of 51 ± 42%. There were triplicate samples of exposure water collected on day 0 of the LWO-Mid treatment that yielded a range of PSD from 11.5% for C1-naphthalene to 70.2% for chrysene with an average of 30.5 ± 21.9% for TPAH and different individual PAH. The RPD ranged from 0.14 to 15.8% for all the PAH and conditions where there were duplicate samples taken. When considering the variation in both water and tissue PAH concentrations, a factor of 2 between the modeled and measured values was considered reasonable. Thus, the simulations are expected to reasonably represent the exposure of the herring eggs.

### Comparison of exposure using simulated toxicokinetics

If the data had been more abundant both temporally and across treatments, direct comparisons could have been made. However, given that toxicokinetics for individual compounds are likely constant across the treatments, based on the above evaluation, and that the simulations reasonably reflected the measured data, expected exposures across all treatments were compared based on the modeling, focusing on phenanthrene, C1-phenanthrenes, dibenzothiophene, and C1-dibenzothiophenes. The High, Mid, and Low concentrations for these PAH were examined for both the LWO and MWO experiments as these were the conditions under which Carls et al. (1997, 1999) observed a wide range of toxicological responses.

**Figure 6:**
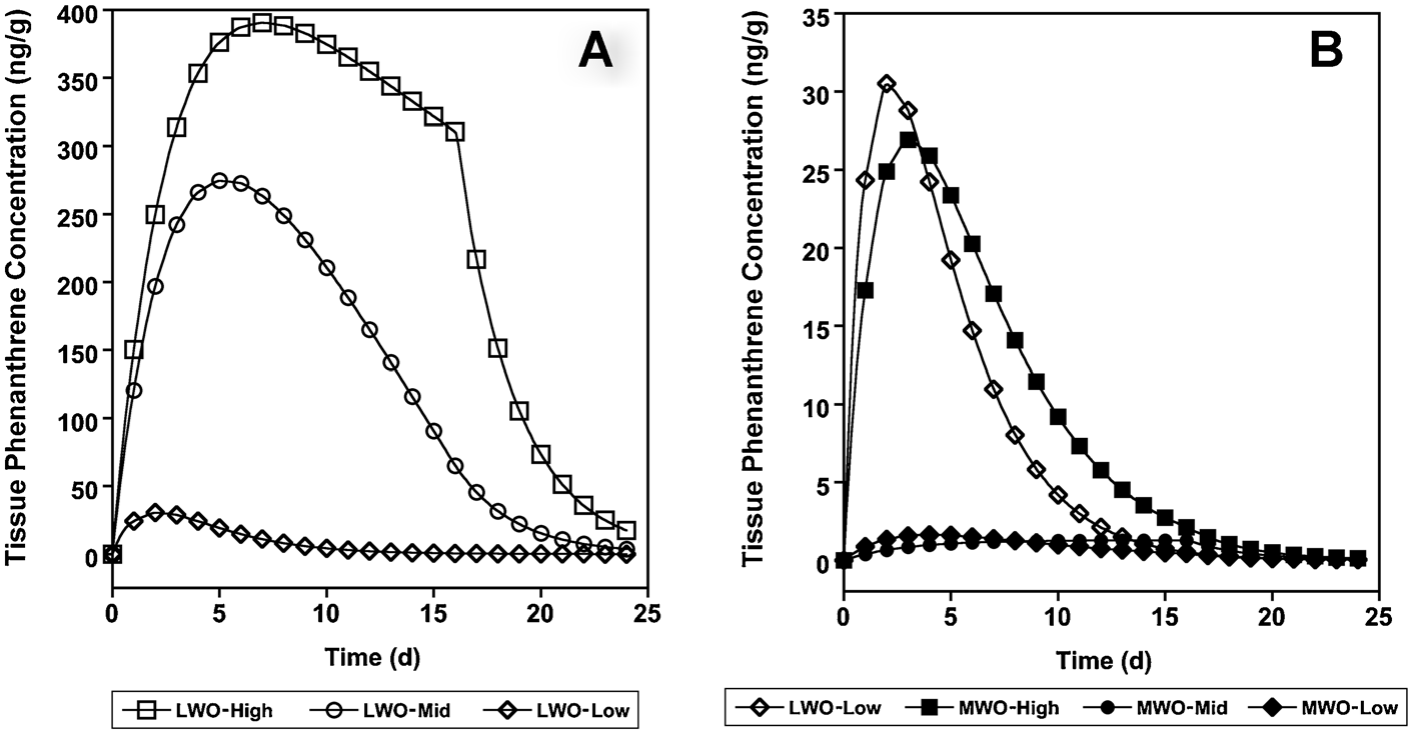
Simulation of exposures of herring eggs to phenanthrene across treatments (**A** and **B**).

Based on the exposure of the herring eggs across treatments to phenanthrene (Figure 6), it is clear that, whereas the toxicokinetic parameters were constant, the exposure conditions led to different predicted temporal patterns and concentrations of phenanthrene in tissues. Specifically, the predicted peak tissue concentration shifted from day 9 for LWO-High (Figure 6A) to approximately day 2 and 3 for LWO-Low (Figure 6A) and MWO-High (Figure 6B), respectively. The LWO-Low and MWO-High treatments had similar exposure patterns although tissue phenanthrene appears to have been eliminated faster in the LWO-Low treatment, reflecting a more rapid decline in the water concentrations. Thus, for phenanthrene exposure to be evaluated at the peak exposure concentration, day-2 data would have been needed for some of the exposures; however, Carls et al. (1997, 1999) took their first tissue sample on day 4 of all treatments except MWO-Mid and, thus, were unable to measure the peak tissue concentration. Tissue samples were collected on days 1, 2, 4, 8, and 16 of the MWO-Mid treatment; tissue phenanthrene concentrations were similar on days 1, 2, and 4 (7.9, 8.0, and 9.9 ng/g dry wt, respectively) and were only slightly higher than in day 2 MWO-Control embryos (7.6 ng/g). Thus, tissue phenanthrene concentration reached a peak between day 2 and 4 in the MWO-Mid treatment. The low frequency of tissue sampling also precluded measurement of the peak tissue concentration of phenanthrene for the LWO-High treatment. This example illustrates the need for frequent sampling when comparing across treatments with different source-loss rates, particularly so that peak concentrations and trends are adequately measured. The fact that tissue concentrations peaked at different time intervals suggests that integrated exposures, even with similar peak concentrations, can occur at different developmental points in early life stages of aquatic animals, such as the different embryonic and larval stages of herring.

Depending on the endpoint and its timing relative to the peak exposure time, magnitude, and mechanism of action, exposures that peak at different times, even if the peak is of the same magnitude, may lead to different responses because of differences in time for repair processes to take place. When both peak tissue concentration and timing differ, particularly for different ingredients in a complex mixture, such as weathered petroleum, determination of the causes of different lethal and sublethal endpoints will be difficult if not impossible. However, estimating exposure based on peak-tissue concentration or the maximum concentration attained within the exposure time selected for the response is expected to lead to a reasonably conservative estimate of the maximum expected response. This expectation assumes that the tissue residue is proportional to the concentration at the site of toxic action and that the substance that is measured is the toxic agent or proportional to it. Even if a specific metabolite of the parent substance is the toxic agent, it should be proportional to the accumulated dose assuming that biotransformation rate is first order within the range of exposure conditions.

C1-phenanthrenes, dibenzothiophene, and C1-dibenzothiophenes (Supplemental Figures 8–10) show a similar variation in the timing of the peak concentration, depending on the source-loss kinetics. The modeled earliest peak tissue concentration for these PAH occurs at day 4, but some of the peak concentrations occur much later and, for C1-phenanthrene LWO-High, the modeled data reflect the measured relatively constant-exposure concentration that varied between 0.7 and 1.1 µg L^−1^ during the 16-day exposure. Measured tissue residues of C1-phenanthrene declined by approximately 38% between day 4 and day 15 in the LWO-High exposure, possibly reflecting induction of CYP1A enzymes that occurs on approximately day 7 postfertilization in Pacific herring embryos (Incardona et al. 2009). These comparisons illustrate the fact that concentrations of different substances peak in the organism at different times after the beginning of the exposure, dependent on both the source kinetics and the toxicokinetic parameters for the particular substance. This fact complicates interpretation of exposure conditions that lead to a toxic response. Even if time-variable response data were available to provide an estimate of toxic equivalents, the fact that timing of uptake varies along with concentration creates a difficult, if not impossible, condition for interpreting mixture interactions for the observed responses. Clearly, data collected from testing, such as conducted by Carls et al. (1997, 1999), are specific to those test conditions and generally cannot and should not be applied to other test conditions or field data without specific consideration of the exposure dynamics.

Complexity increases when considering TPAH rather than individual PAH in the complex mixture, because the proportion and concentration of the PAH in the mixture contributing to the observed response vary along with the variation in peak concentration timing and intensity (Supplemental Figure 11). What appears as a set of time-variable kinetics is actually the summation of the kinetics of the individual substances that make up the particular mixture and the individual decline in source concentrations for each substance at each treatment level.

In each treatment, there is clear variation in source concentration (Table 1). Thus, TPAH do not represent any specific mixture at any point in time in the exposure or among treatments. Even assuming that response to a specific TPAH mixture were known, the variation in the composition and timing of the peak tissue concentrations of the individual components among the treatments would lead to an uneven evaluation of exposures if one selected TPAH as the dose metric based on tissue concentration. This would be the case even accounting for the differences in timing and concentration of the peak value. Modeling of TPAH detailed above largely overestimated exposure (Supplemental Figure 7) because of both differences in the contributions of the various substances in the mixture to the TPAH profile and the use of estimated values from the LWO-High treatment. Clearly, TPAH should not be used as a dose metric in either water or tissue. This complicates the interpretation of the response data and indicates the need for investigation of specific causative agents and the temporal thresholds for such responses. Combining the impact of multiple compounds in mixtures through approaches such as a toxic unit analysis would help focus on both individual compound potency and would assist in sorting out compounds acting by different mechanisms of action.

## CASE STUDY 2: STATIC AND STATIC RENEWAL EXPOSURES

Many aquatic toxicology studies are performed under either static or static-renewal conditions. Under static conditions, the concentration of the bioavailable fraction of the test substance in the exposure water can be depleted by processes such as volatilization, sorption to the surfaces of the exposure container, degradation, precipitation or coalescence into droplets, and accumulation by the test organism. The physical characteristics of all the chemical components of the test substance, physical chemical loss processes, the substance concentration, organism to water mass ratio for the system and the toxicokinetics dictate the rate of change in concentration of the test substance in the exposure water. The rate of change of the concentration in water in combination with the organism toxicokinetics should have a similar effect on the bioaccumulated dose as described above for the first case study—except that the organism will come to equilibrium more rapidly because eliminated test substances, as well as any transformation products, become available for re-uptake. In fact, if the only loss processes result from bioaccumulation, the organism will show bioaccumulation that looks similar to constant exposure experiments except for the magnitude of the accumulation. The magnitude of accumulation will depend on the water volume to organism mass ratio, with lower accumulation with lower ratios.

When we began this review, we initially considered focusing on complex petroleum mixtures with a second case study on WAFs. Unfortunately, we could not find published studies with either a static or static renewal WAF of crude or refined oil that had repeated quantitative measures of total or individual petroleum PAH concentrations in both exposure water and tissues of test animals over the course of the exposure. Thus, we could not fully investigate how changes in the loss rate of different chemicals in the exposure mixture affected the rate of uptake and maximum accumulated tissue dose. However, there are studies using other nonpolar test substances that provide insights in this regard. To this end, we selected a study of a single compound, dieldrin, that demonstrates the principles that govern the static exposure conditions for nonpolar organic contaminants where the water concentration changes; changes in the characteristics of the compound will govern the toxicokinetics and therefore the change in water concentration, all other variables being equal. Discussion of other exposure conditions is similarly limited in the types of compounds used and examples available; as such, the exposure to pentachlorophenol was chosen to demonstrate exposure conditions with the understanding that principles controlling the toxicokinetics of nonpolar organics are similar among compounds.

### Static exposures

**Figure 7:**
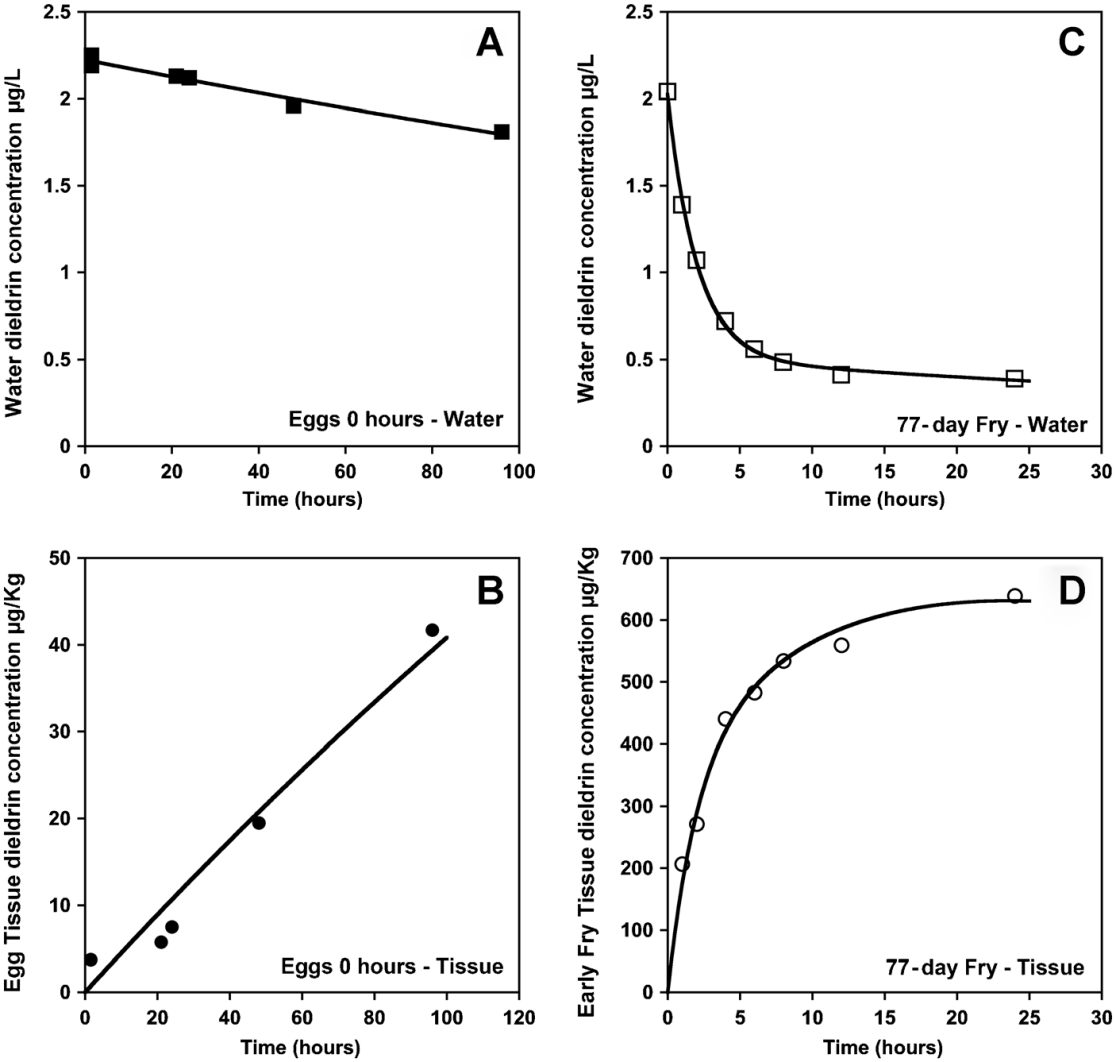
Water and tissue kinetics for the static exposure initiated at day 0 postfertilization for rainbow trout eggs (**A** and **B**) and for fry 77 d postfertilization (**C** and **D**) to ^14^C-dieldrin. Based on interpolation of data given in Van Leeuwen et al. (1985). Data for water followed a single exponential decay for (A) and a double exponential decay for (C). The tissue kinetics (B and D) follow the first order uptake and elimination model (Eqn. 1).

As a case study where study where toxicokinetics and toxicity were both determined under static conditions, we use the study of Van Leeuwen et al. (1985), that tested the toxicity of dieldrin, a nonpolar organic contaminant, using a static-renewal experimental design, and examined the toxicokinetics by measuring the uptake of ^14^C-labeled dieldrin by rainbow trout (*Oncorhynchus mykiss*) eggs and fry under static conditions. Exposure of eggs and fry was initiated at several times postfertilization and the toxicokinetics were determined for exposures ranging from 24 to 190 h, depending on life stage (Figure 7). Both tissue and water dieldrin concentrations were determined. The decline in water concentration varied with the early life stage investigated. Using data estimated from the figures in Van Leeuwen et al. (1985), the loss rate from water was first order for all cases except for the early fry 77-d stage, where a double exponential was required to fit the decline in water concentration. For eggs, the decline in water concentration had a half-life of approximately 309 h for bioconcentration tests with newly fertilized eggs (Figure 7A) to approximately 136 h for tests with late-eyed eggs. For fry, the water concentration declined more rapidly, with a half-life of approximately 44 h for sac fry 42 d postfertilization to 1.4 h for early fry 77 d postfertilization (Figure 7C). The short half-life for early fry occurred despite a large volume to mass ratio selected for the experiment because of the rapid toxicokinetics at this stage. The combination of conditions yielded a lower dieldrin concentration in tissue than for the other stages. In all cases, the eggs and fry had uptake curves demonstrating a smooth accumulation toward a steady state (Figures 7B and 7D; see also Figure 2 in Van Leeuwen et al. [1985] for additional life-stage details). This smooth accumulation is a result of the balance between uptake and elimination when the eliminated compound is immediately available for re-uptake and essentially the mass of contaminant resides in the water and organism in proportion to the capacities of the 2 media.

Van Leeuwen et al. (1985) determined toxicokinetics using a mass-balance approach, which leads to system-dependent rate constants (Landrum et al. 1992). Sufficient information was provided to allow calculation of concentration-based uptake-rate constants using the bioconcentration factor values provided and the elimination-rate constant, which is not a system-dependent value. This was accomplished by using the relationship between the bioconcentration factor (*BCF*) and the elimination-rate constant to calculate k_u_ from the following equation, k_u_ = *BCF* × k_e_. The uptake-rate constants ranged from 0.22 L kg^−1^ h^−1^ for freshly fertilized eggs to 141 L kg^−1^ h^−1^ for early fry. The 0-h and 24-h postfertilization egg stages had similar uptake-rate constants (Van Leeuwen et al. 1985), whereas uptake rate constants increased for 14- to 28-d postfertilization eggs. The fry had much higher uptake-rate constants (Figure 7D). The eggs at all stages had similar elimination-rate constants, yielding tissue residue half-life values of 15 to 19 d; the sac fry had a half-life of 22 d, and the early fry a half-life of 0.3 d. These rates had a bearing on the toxicity observed for 96-h exposures to dieldrin under a separate static-renewal experiment reported by Van Leeuwen et al. (1985). Despite initiating the exposures at similar concentrations, the accumulated dose at the end of the exposure varied reflecting the differences in both the toxicokinetics and the organism mass to volume ratios. Saturated solutions of dieldrin were not acutely toxic to eggs and sac fry; however, the early-fry stage was sensitive (96-h LC50 = 3 µg/L), which may have resulted both from a more than 10-fold increase in uptake rate, compared to the eggs and yolk fry, and from the onset of biotransformation of dieldrin to the more toxic aldrin-transdiol (Sudersham and Khan 1981).

When 24-d old eggs were exposed for 48 h to 5.8 µg/L dieldrin, 79% of the bioconcentrated dieldrin was in the yolk sac and 15% was in the body of the embryos. At hatching, dieldrin concentration declined in the yolk and increased in the body of the sac fry. The calculated critical tissue concentration in the larvae was 5 µg g^−1^ in early fry and 36 µg g^−1^ for the sac fry. Thus, mobilization of yolk lipids and the onset of biotransformation in the fry are critical to the observed toxicity. This also sets the timing of the onset of dieldrin biotransformation capability for this species. The one drawback to Van Leeuwen et al. (1985) is that the toxicokinetics determined by the ^14^C-labeled dieldrin experiments were not measured under the same exposure concentrations as in the toxicity tests.

There is an absence of studies that track both changing water concentration and tissue concentrations for exposure to petroleum WAF. Barron et al. (2003) developed some of this information when they exposed Pacific herring eggs and larvae to a WAF of weathered Alaska North Slope crude oil in the presence and absence of sunlight. The larvae were exposed under static conditions for 24 h; 96-h static renewal conditions were used for eggs. Toxicity was assessed based on TPAH concentration, measured either as the initial aqueous concentration or tissue concentration after a 4-d exposure of embryos or a 1-d exposure of larvae. There was no attempt to trace the dynamics of the exposure and bioconcentration for either of the 2 life stages; however, toxicity to the embryos was followed beyond the actual exposure period to examine latent toxicity. Changes in the PAH concentrations and chemical compositions of the exposure mixtures were observed; however, actual causal agents of toxicity were not identified. The dose metric (aqueous TPAH concentration) selected for assessing response was not appropriate because of the changing PAH exposure concentrations and compositions, particularly in the presence of sunlight. Peak tissue concentration of PAH was likely the most accurate measure of exposure and effective dose (Landrum et al. 2012).

There is an absence of data sets for mixtures such as petroleum fractions dissolved and/or dispersed in water that monitor the absorbed dose, the exposure dynamics, and the response of organisms in static or static renewal exposures. This is a surprising deficiency that limits our ability to interpret the interactions of exposure dynamics and toxicokinetics on the toxic response to complex mixtures. The chemical exposure concentration and composition and the amount of absorbed dose will vary with the organism mass-to-water-volume loading rate for static or static-renewal exposures when organisms are exposed at the same concentration. Furthermore, different developmental life stages of test organisms will have different toxicokinetics and different toxicological responses, particularly when biotransformation is critical to the toxicological endpoints. Thus, studies are needed that examine these variables along with toxic responses to provide the time-dependent thresholds important for assessing responses to varying exposure concentrations.

### Pulsed (static-renewal) exposures

Static-renewal exposures are special pulsed exposures where the exposure-medium replacement is at sufficiently frequent intervals to minimize the substantial decline in water concentration found in static exposures. These experimental designs result in a time-weighted average exposure that simulates a constant-exposure environment if the elimination rate of the test substance is slow and the renewal frequency is high (Landrum et al. 2004). However, when the replacement frequency is low relative to the elimination rate of the test organism, the dynamics of exposure begin to affect the absorbed dose, and both the toxicokinetics and toxicodynamics become critical to the response of the organism.

In one of the earliest studies of pulsed exposures, the toxicity of pentachlorophenol to larval fathead minnows was explored in terms of the role of pulse duration and pulse frequency using the exposure water concentrations and estimating the critical tissue-residue concentrations, based on the toxicokinetics from a separate bioaccumulation test which were confirmed from the toxicokinetics determined from the toxicity tests, as the dose metric to produce mortality (Hickie et al. 1995). Water concentrations, based on summed exposure duration, and by extension the time-weighted average exposure concentration, were not constant across the various exposure scenarios explored. Specifically, the concentration required to produce the 96-h LC50 declined with increasing exposure duration to an incipient level of approximately 1 µmol L^−1^ for long-term exposures even with intervals of 24 h between pulses. A better dose metric across the variety of exposures was the estimated critical body residue required to produce a toxic response. The estimated absorbed dose associated with 50% mortality was nearly constant. However, the critical body residue was not confirmed with actual measured residues. The Hickie et al. (1995) study demonstrates the difficulty of assessing toxicity, under pulsed exposure conditions, based only on the measured water concentration, even when water concentrations are measured frequently.

Zhao and Newman (2004, 2006) demonstrated that toxicity continues after pulsed exposure has ceased. Thus, during pulsed exposures, both the toxicokinetics and toxicodynamics should be considered to best understand the observed response. Models developed to include this concept hypothesize that there is the formation and repair of damage, which describe the temporal process for the toxicodynamics when the production of damage is driven by the amount of absorbed dose (Lee et al. 2002). Models of this type have been improved and applied to pulsed exposures (Ashauer et al. 2006). These models have been refined such that there is now a general, unified threshold-model for survival for use in ecotoxicology (Jager et al. 2011) and a companion general approach for quantal and graded sublethal endpoints (Ashauer et al. 2011). The general drawback to these models is that they require a large amount of data for application. However, when exposures are dynamic, proper interpretation of the effects of the exposures will require this level of detail, including frequent measures of exposure conditions, the absorbed dose and, if possible, the extent of response. If these models are to be applied in the field to appropriately address complex mixture exposures, substantial efforts will need to be made to determine the overall kinetics, including biotransformation, for the species and life stages of interest as well as temporal threshold response data to permit appropriate interpretation to dynamic-exposure conditions. A recent review of the toxicological effects of episodic exposure provides similar conclusions not only for organic contaminants but also for metals (Gordon et al. 2012). Furthermore, there is a strong suggestion from that review that not only peak tissue residue but also total exposure duration will be critical to the evaluation, which is clearly part of the unified threshold model put forward by Jager et al. (2011).

## LESSONS LEARNED FROM THE THEORETICAL SCENARIOS AND THE TWO CASE STUDIES

The following lessons follow from the theoretical scenarios and the case studies for exposure to dynamic conditions:

- Initial water concentrations provide an estimate of maximum exposure, are generally not useful exposure measures for highly dynamic conditions. Reliance on initial water concentrations will introduce greater uncertainty in the conclusions when comparing between laboratory experiments, laboratory and field conditions, or field sites with varying exposure dynamics.
- Geometric means of water concentrations can provide misleading information regarding aqueous exposure conditions relative to peak accumulated concentration and dynamics of exposure and therefore toxicity endpoints—unless the rate of change of concentration is small. Arithmetic means (Carls et al. 2005) will be similarly flawed. The conditions where the application of averages can be safely applied still need to be determined.
- Interpreting the toxicity of a substance using the peak tissue concentration is likely endpoint specific and, without time specific threshold values, can also be misleading and problematic. However, peak tissue residues provide a maximum exposure and are expected to provide a better dose metric than water concentrations as they account in large part for the dynamics of exposure.
- Frequent sampling of both water and tissue is essential when comparing across treatments with differing source-loss rates so that peak concentrations and trends are adequately measured. This is particularly important for rapidly developing early life stages, such as fish embryos, and for dynamic exposure conditions.
- Water and tissue dose metrics should be based on individual substances, not on complex mixtures, such as TPAH, because the dynamics of the individual compounds in the mixture may vary, leading to unequal representation of potency when using the mixture as the dose metric. To address mixtures, approaches such as toxic unit analyses will assist in addressing the relative potency of mixture components as well as helping address differences in the mechanism of action. Thus, having temporal thresholds for application of this approach will be important for application to field conditions.
- Extrapolation between different test and exposure conditions should be done cautiously. When considering the results of a mixture study under dynamic conditions (Case Study 1), it is likely difficult, if not impossible, to extend the results for such dynamic exposures beyond the particular experiment, given the present state-of-knowledge on the dynamic toxicity of compounds. An example of this comes from oiled-gravel-column studies in which pink salmon (*Oncorhynchus gorbuscha*) embryos were exposed to PAH-containing column effluent in 2 different experiments (Heintz et al. 1999; Carls et al. 2005) as described in Page et al. (2012). Peak concentrations and compositions of TPAH differed in both magnitude and timing between the 2 experiments (Figure 3 in Heintz et al. 1999; Figure 1B in Carls et al. 2005), possibly related to different loading and flow rates among the gravel columns for the 2 experiments, which would have affected the exposure dynamics and thus the PAH uptake and the toxicodynamics and the resultant relationships between exposure and effects.

## CONCLUSIONS

We compliment the authors of the 2 case studies for the extent of data provided, which allowed for the analyses and *Lessons Learned* above. Clearly other authors need to provide more complete data sets and adequately analyze those to fully relate exposure of specific mixture components to observed responses and to determine causation. The dynamics of all mixture components need to be considered—not just a simple summation of those components that too often lead to incorrect conclusions.

The above *Lessons Learned* apply not just to organic substances and mixtures of those substances but also to inorganic substances such as metals and mixtures of those substances. Adams et al. (2011) provide a useful review of the relationship between metal body burdens and toxic responses.

There is a pressing need to develop exposure–duration response data such as Frantzen et al. (2012) attempted for pyrene using a dynamic–response model (Kooijman and Bedaux 1996). Although their work did not resolve the confounding issue of exposure dynamics for pyrene, it did advance our understanding of pyrene exposure–response relationships. We encourage continued development of temporal response concentration relationships focusing on tissues, not water (Landrum et al. 2004; Schuler et al. 2004; McCarty et al. 2011) so that tissue concentrations can be applied to develop temporal thresholds for response. The development of these temporal relationships can be helped by methods that ensure constant exposure, as in the work of Engraff et al. (2011), which involved passive dosing of PAH compounds and mixtures. Once we fully understand constant exposures, we will be better able to understand the more common reality of pulse exposures.
