# Supplementary material for: Influence of Exposure and Toxicokinetics on Measures of Aquatic Toxicity for Organic Contaminants: A Case Study Review
Source: Integr Environ Assess Manag. 2012 Dec 10;9(2):196–210. doi: 10.1002/ieam.1388 (PMC3664022; doi:10.1002/ieam.1388)
Supplement: Supplementary file 1 [file ieam0009-0196-SD1.pdf]

Supplementary figures referred to in the text. All tissue concentrations are on a wet tissue weight basis.

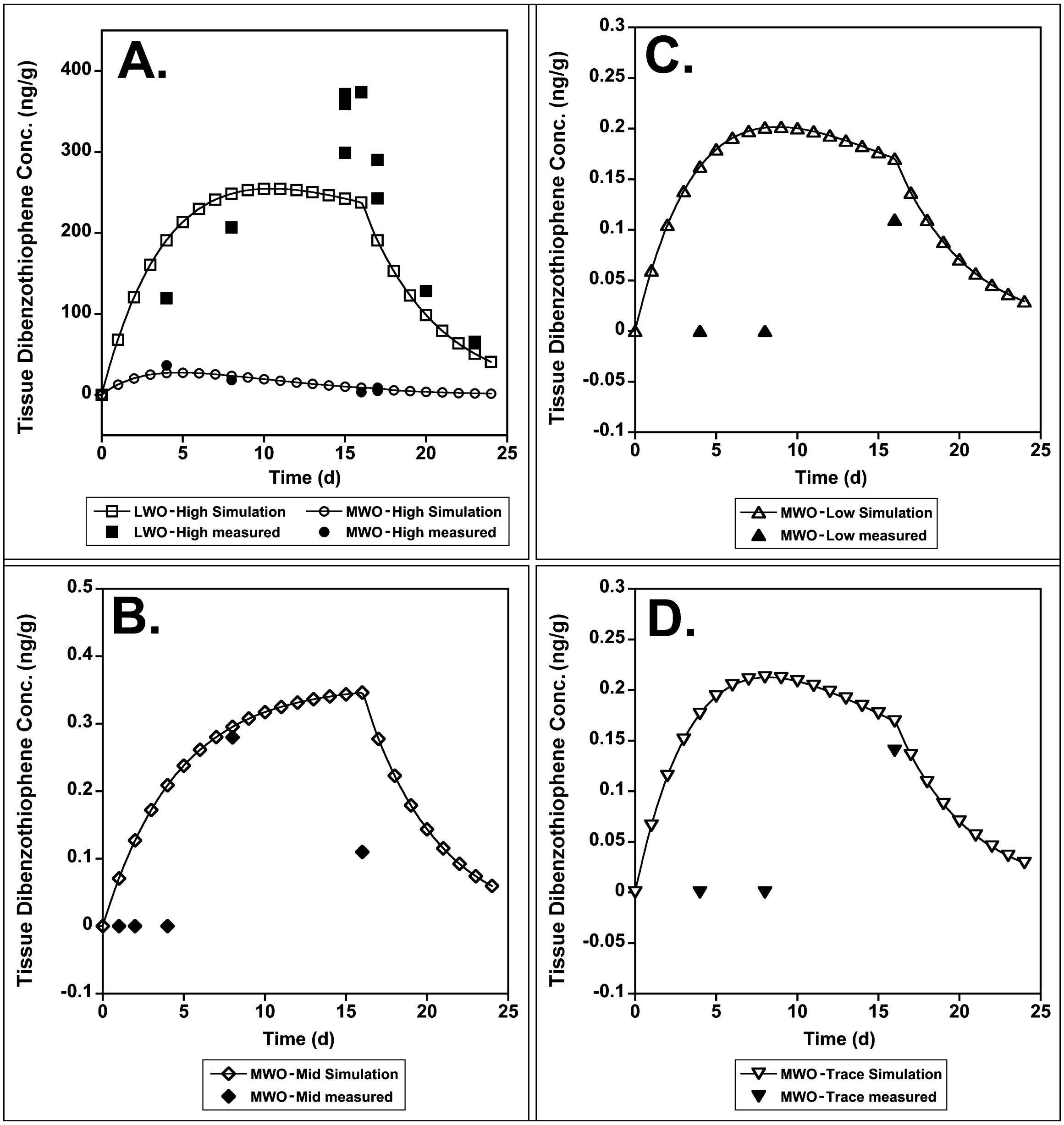

Figure S1 A-D. Comparison of the toxicokinetic model results with measured tissue data for dibenzothiophene across all treatments .

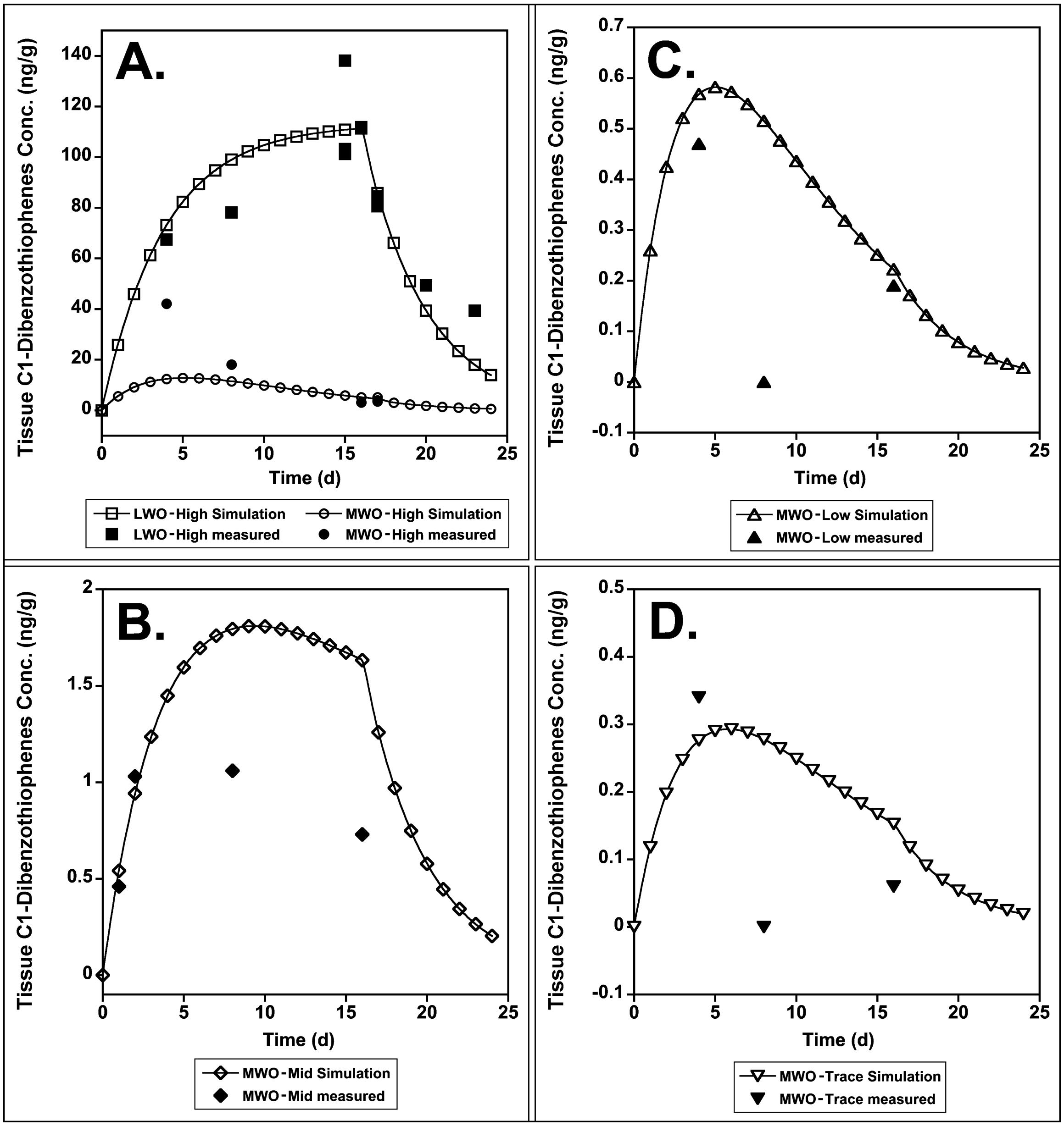

Figure S2 A-D. Comparison of the toxicokinetic model results with measured tissue data for C1-dibenzothiophenes across all treatments.

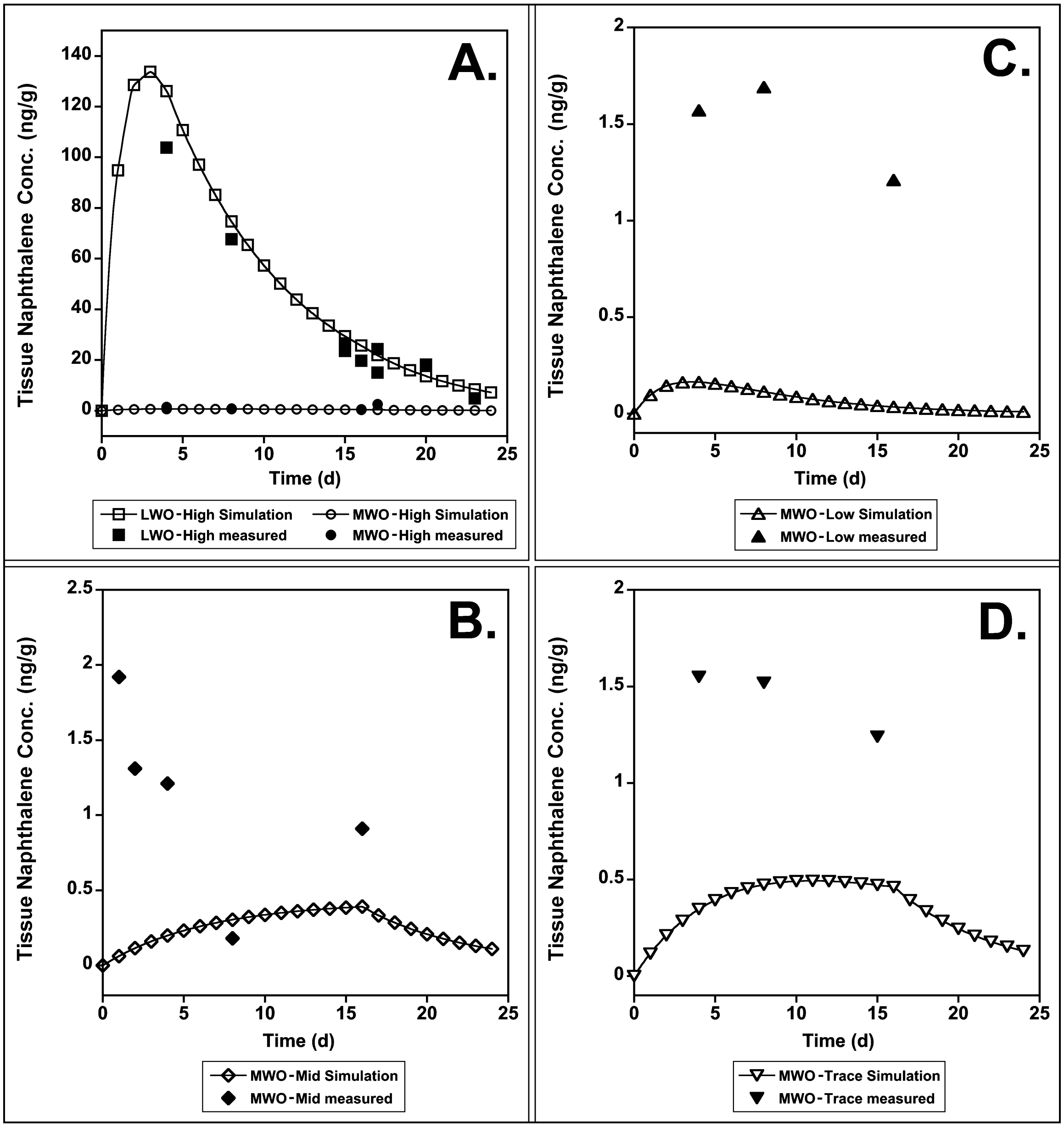

Figure S3 A-D. Comparison of the toxicokinetic model results with measured tissue data for naphthalene across all treatments.

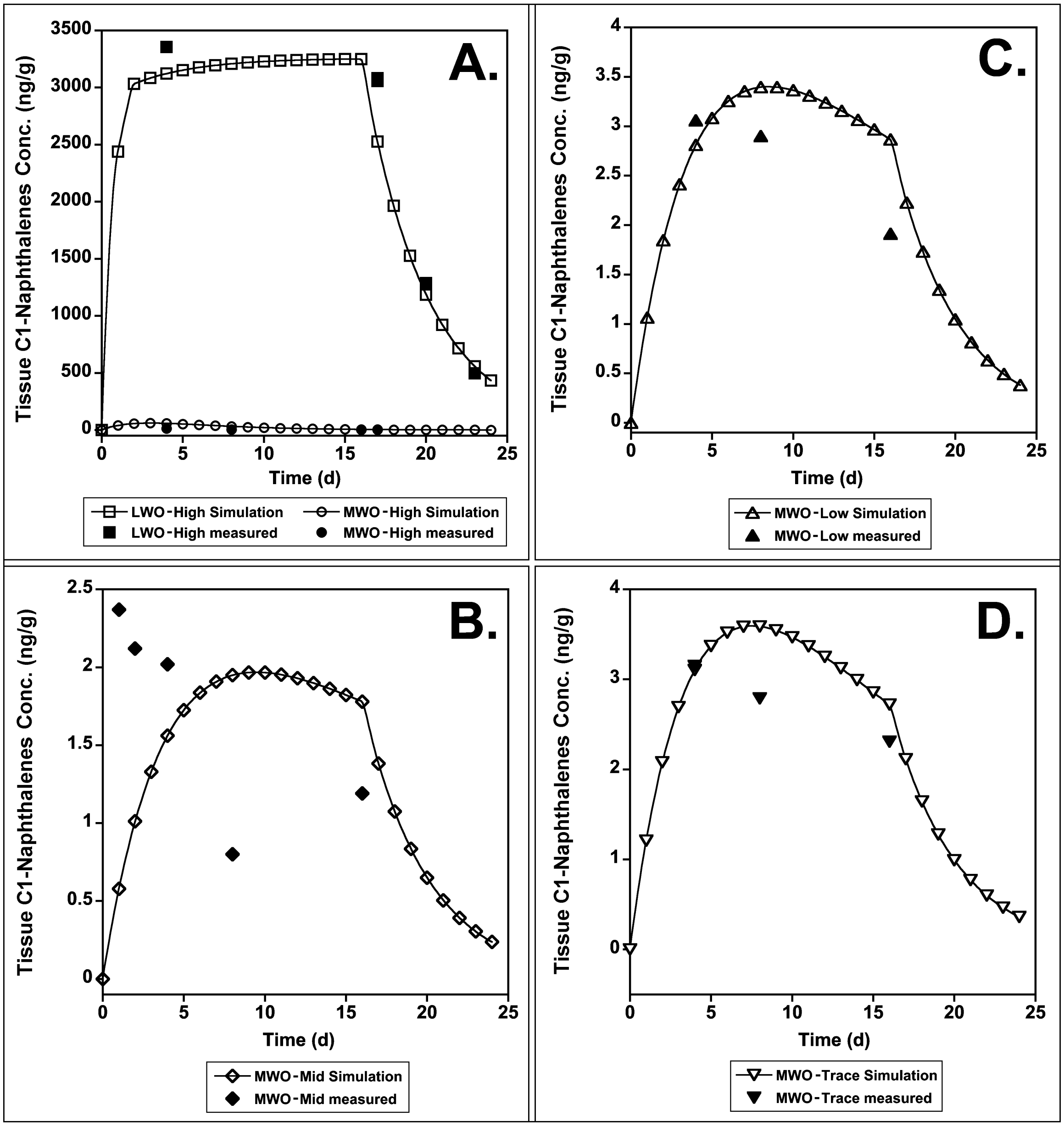

Figure S4 A-D. Comparison of the toxicokinetic model results with measured tissue data for C1-naphthalenes across all treatments.

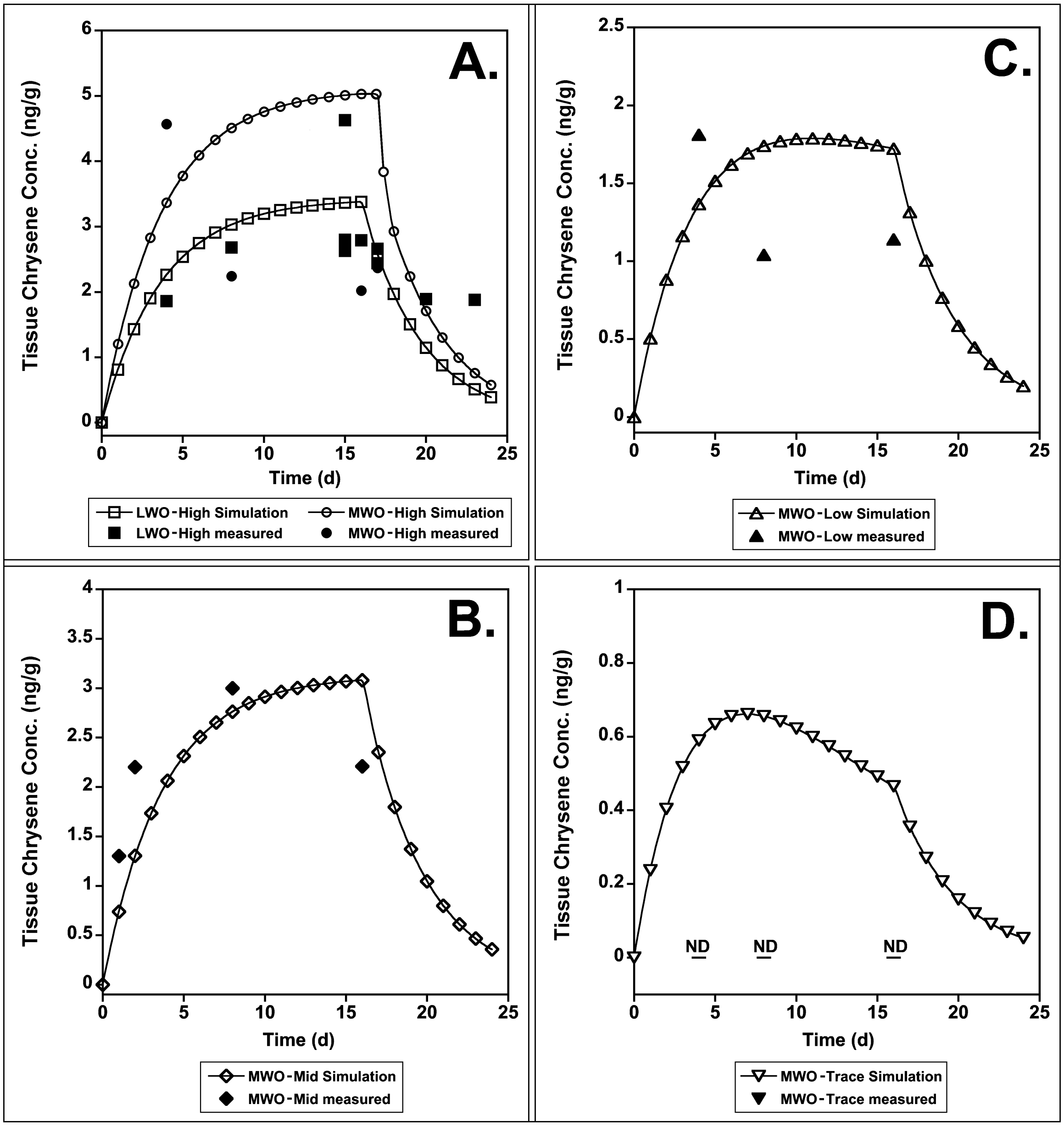

Figure S5 A-D. Comparison of the toxicokinetic model results with measured tissue data for chrysene across all treatments .

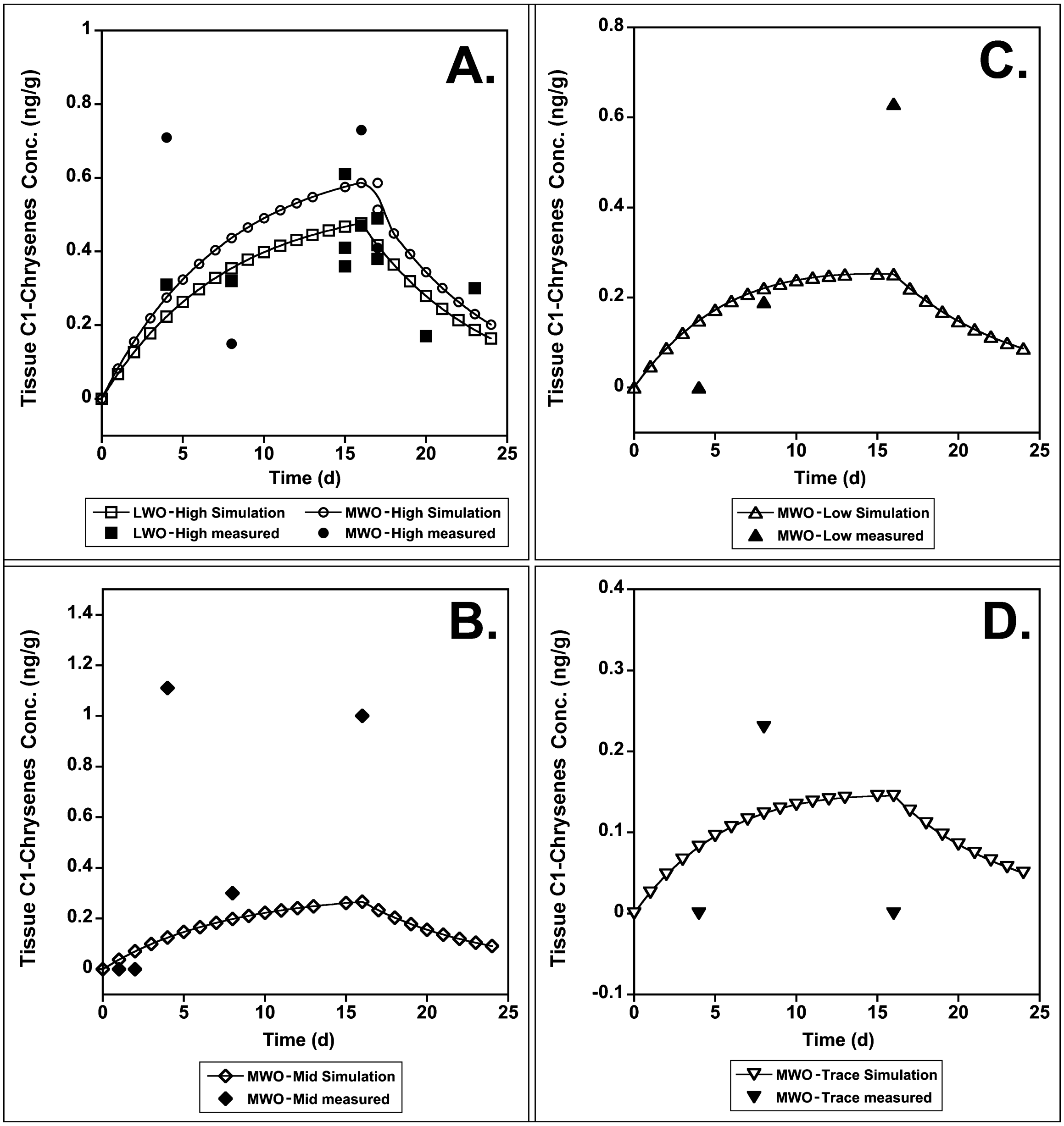

Figure S6 A-D. Comparison of the toxicokinetic model results with measured tissue data for C1-chrysenes across all treatments.

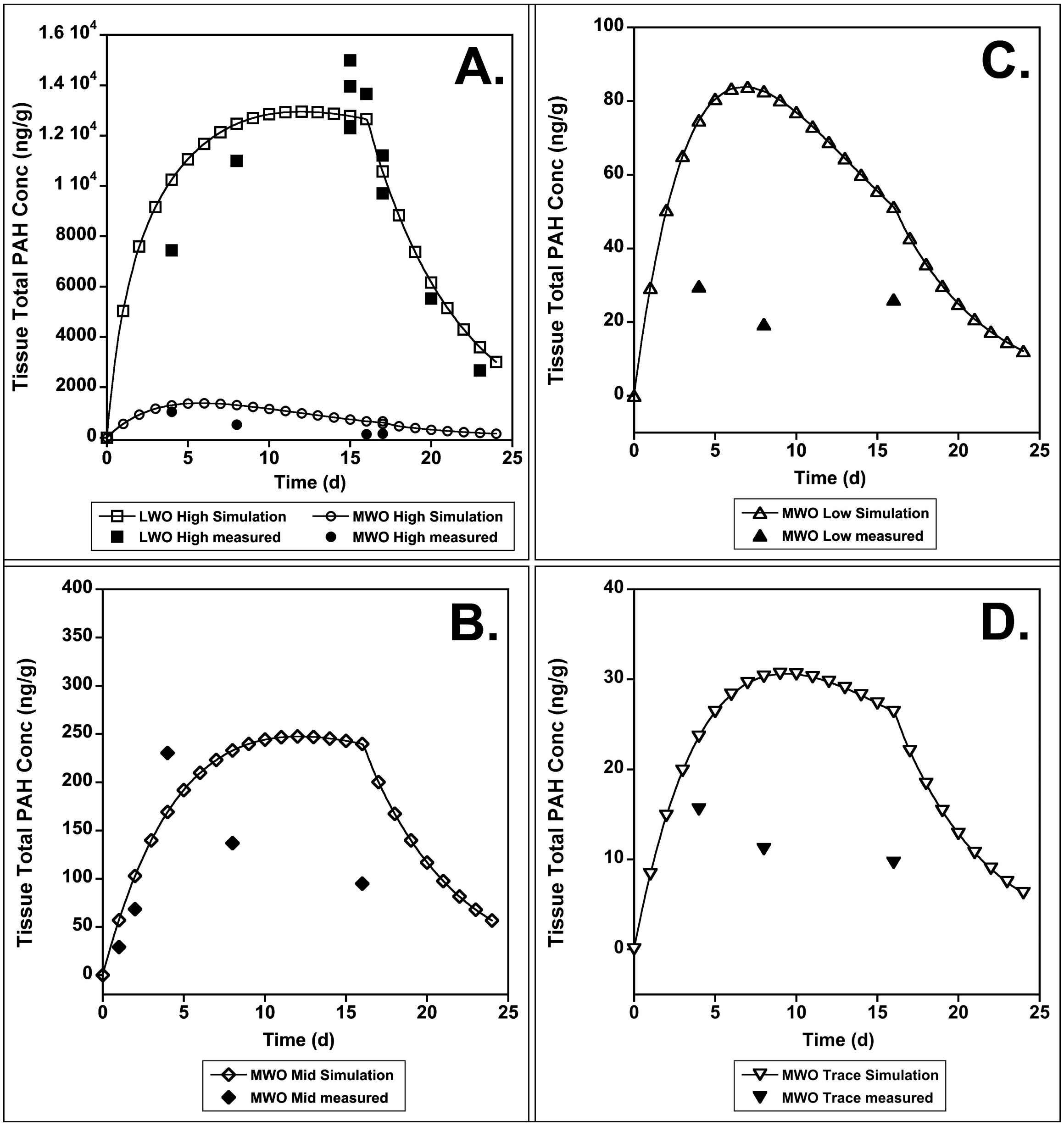

Figure S7 A-D. Comparison of the toxicokinetic model results with measured tissue data for TPAH across all treatments.

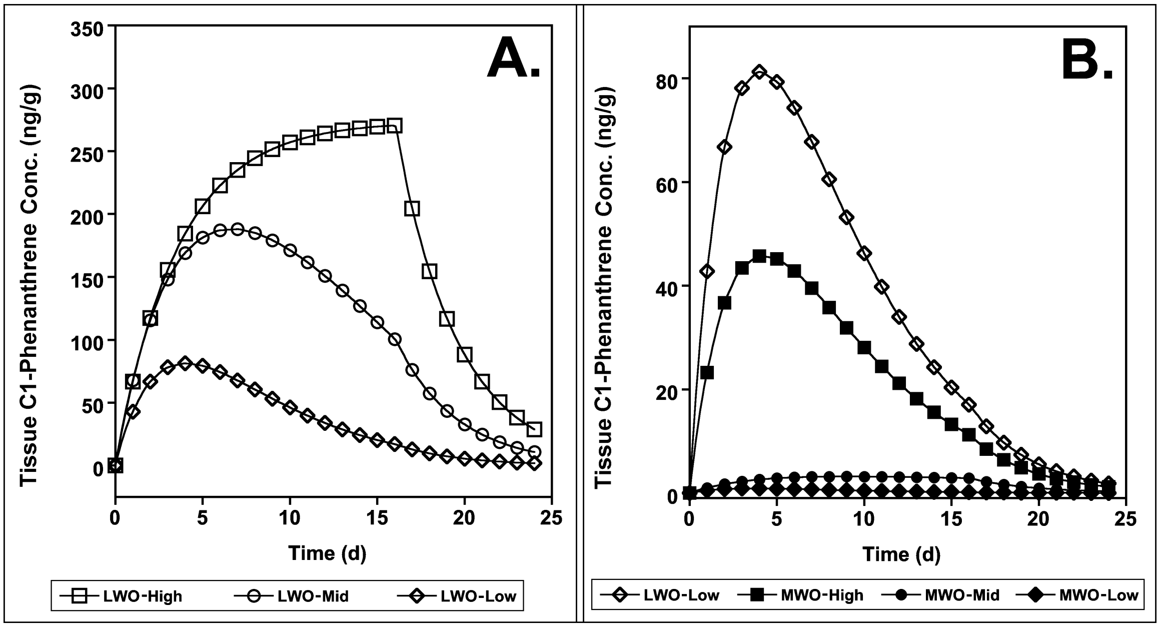

Figure S8 (A-B). Simulation of exposures of herring eggs to C1-phenanthrenes across treatments.

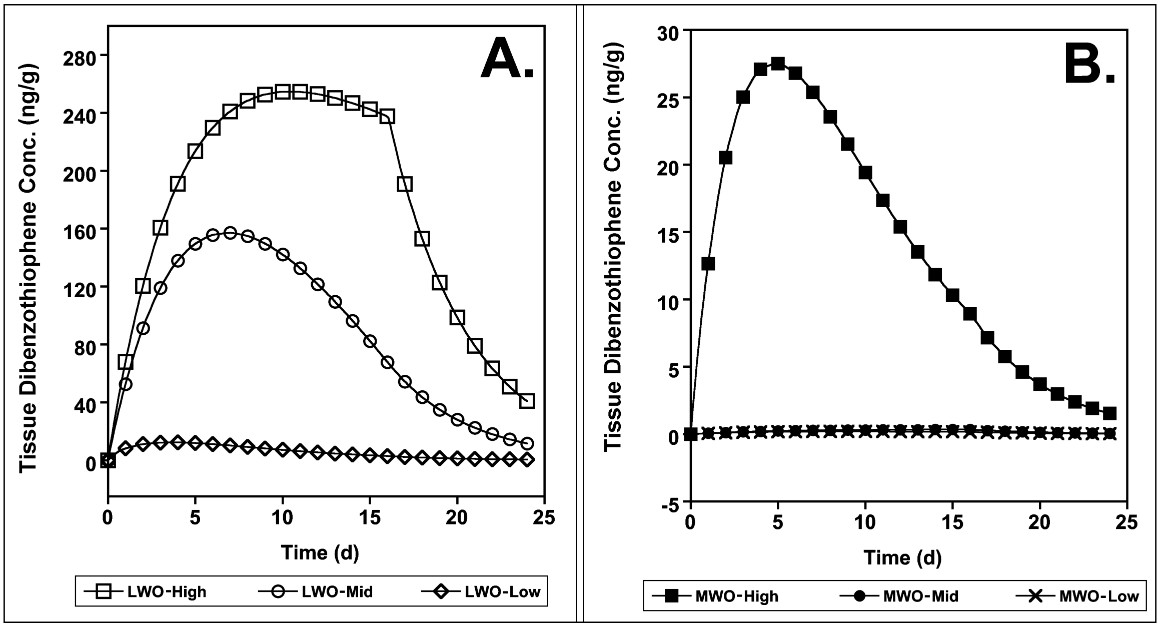

Figure S9 (A-B). Simulation of exposure of herring eggs to dibenzothiophene across treatments.

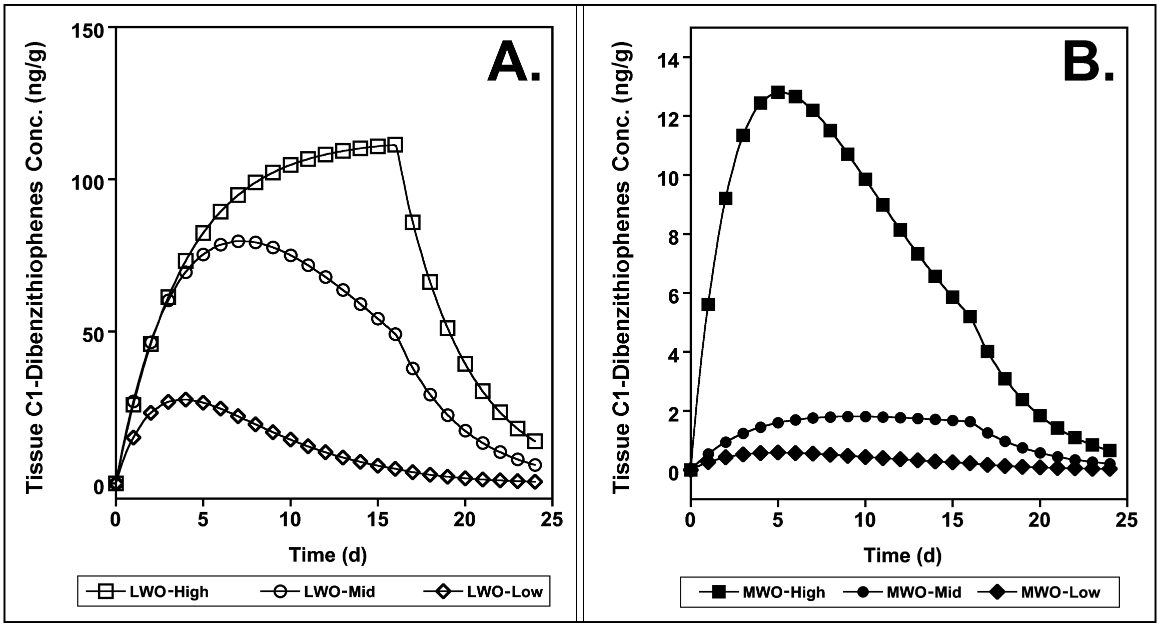

Figure S10 (A-B). Simulation of exposure of herring eggs to C1-dibenzothiophenes across treatments.

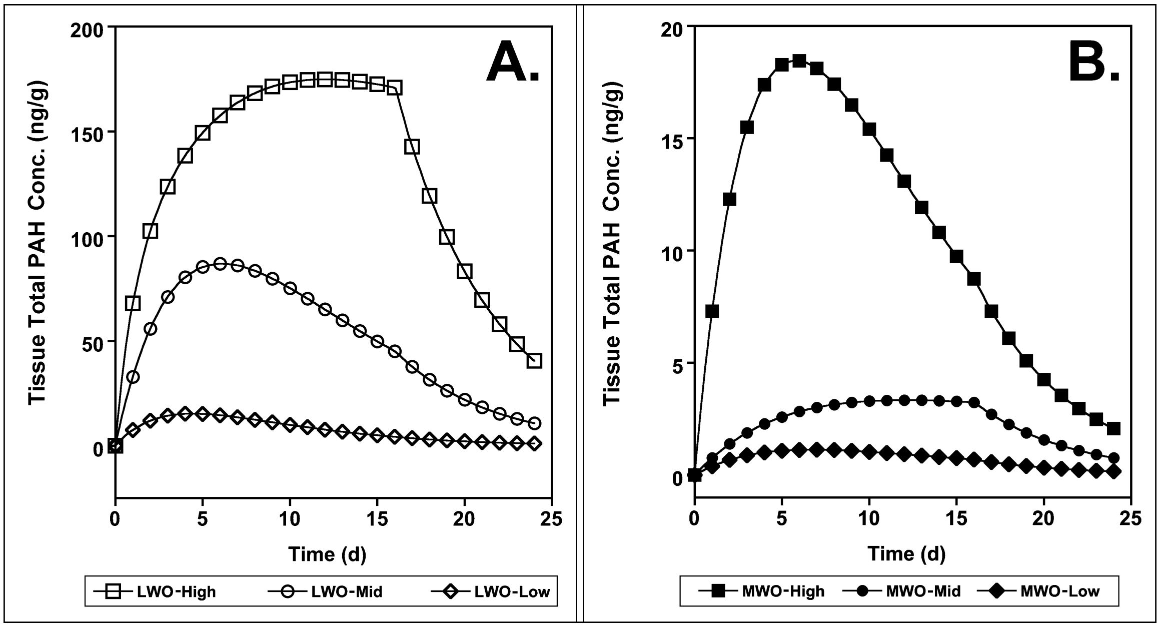

Figure S11 (A-B). Simulation of exposure of herring eggs to TPAH across treatments.
